# Hainanerectamines A–C, Alkaloids from the Hainan Sponge *Hyrtios* *erecta*

**DOI:** 10.3390/md12073982

**Published:** 2014-06-30

**Authors:** Wen-Fei He, Duo-Qing Xue, Li-Gong Yao, Jing-Ya Li, Jia Li, Yue-Wei Guo

**Affiliations:** 1 School of Pharmaceutical Sciences, Wenzhou Medical University, Wenzhou 325035, China; E-Mail: wenfeihe@126.com; 2 State Key Laboratory of Drug Research, Shanghai Institute of Materia Medica, Chinese Academy of Sciences, Shanghai 201203, China; E-Mails: xdq@163.com (D.-Q.X.); liyao24106@hotmail.com (L.-G.Y.); jyli@163.com (J.-Y.L.); jli@simm.ac.cn (J.L.)

**Keywords:** sponge, *Hyrtios erecta*, Thorectidae, hainanerectamines, indole, β-carboline, Aurora A

## Abstract

Two new indole alkaloids, hainanerectamines A (**1**) and B (**2**), and one new β-carboline alkaloids, hainanerectamines C (**4**), along with five known related alkaloids (**3**, **5**–**8**), have been isolated from the Hainan marine sponge *Hyrtios erecta*. The structures of new compounds **1**, **2** and **4** were determined by detailed analysis of their 1D and 2D NMR spectra and by comparison of their spectroscopic data with those of related model compounds. Compounds **2**–**4** exhibited moderate inhibitory activity against Aurora A, a member of serine/threonine kinase family involving in the regulation of cell division and a new target in cancer treatment, with IC_50_ values of 24.5, 13.6, and 18.6 μg/mL, respectively.

## 1. Introduction

Marine sponges of the genus *Hyrtios* (Family Thorectidae, Order Dictyoceratida) have proven to be a rich source of bioactive secondary metabolites, including terpenoids [1,2,3,4,5], macrolides [6,7,8,9,10], and tryptamine-derived alkaloids [11,12,13]. The most important metabolites of the genus *Hyrtios* discovered to date include the powerful anticancer agents, spongistatins **1**–**3** [8,9,10].

**Chart 1:**
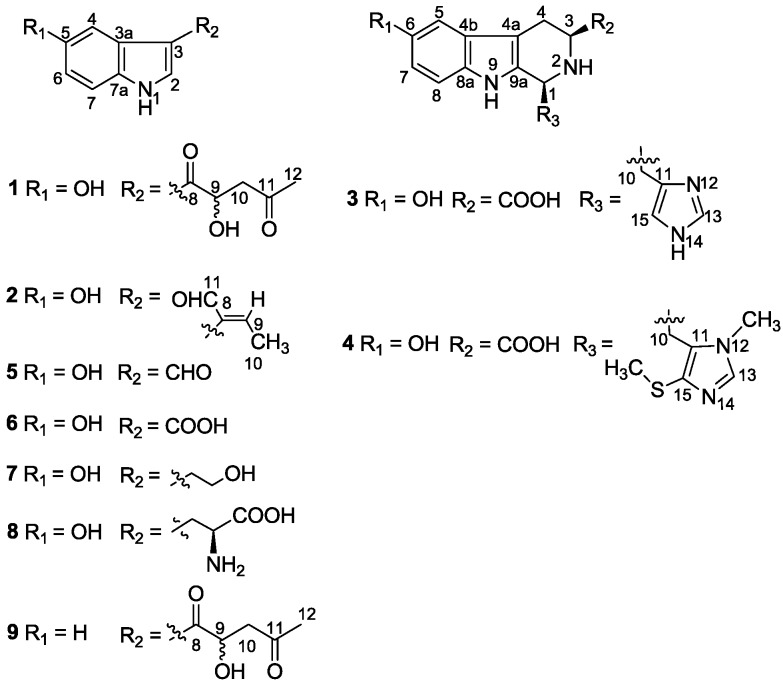
Structures of compounds **1**–**9**.

In our previous paper [14], we reported the isolation of several sesterterpenes including five new ones, hyrtiosins A–E, and two well-known sesterterpenes hyrtiosal [15,16] and scalaradial [17,18,19] from the title sponge. Interestingly, in the course of this study, we had discovered that hyrtiosal could inhibit not only PTP1B (protein tyrosine phosphatase 1B) [20], a promising target in the treatment of type-II diabetes and obesity [21], but also HIV-1 integrase [22]. While scalaradial was found for the first time to be a secretory phospholipase A_2_ inhibitor [23,24]. These findings stimulated our interest to search for more structurally unique and biologically active substances from the sponge. Recently, we re-collected this sponge off the Lingshui Bay of Hainan Province, China, and chemically investigated it. On separation of the Et_2_O- and *n*-BuOH-soluble fractions of an acetone extract of this animal, eight Dragendorff positive compounds, of which three are new, named hainanerectamines A–C (**1**, **2**, **4**), along with five known related indole alkaloids (**3**, **5**–**8**) (Chart 1), were isolated. This paper describes the isolation and structure elucidation of these new compounds.

## 2. Results and Discussion

The usual workup of the Et_2_O- and *n*-BuOH-soluble materials of the acetone extract of the sponge yielded the new alkaloids **1**, **2**, **4**, and five known related analogues **3**, **5**–**8**, as well as previously reported sesterterpenes [14]. The known alkaloids except for **3** were readily identified as 5-hydroxyindole-3-carbaldehyde (**5**) [11], 5-hydroxyindole-3-carboxylic acid (**6**) [25], 5-hydroxy-3-(2-hydroxyethyl)-indole (**7**) [13], and 5-hydroxytryptophan (**8**) [25], respectively, by analysis of their NMR data and by comparison with the data reported in the literature. While the identification of alkaloid **3** as hyrtioreticulin B was not straight forward, it’s a matter to be discussed herein. The NMR data of new compounds **1**, **2**, **4** are strongly reminiscent of those of the co-occurring **3**, **5**–**8**. In particular, interpretation of their NMR spectra [^1^H, ^13^C NMR (Table 1), ^1^H-^1^H COSY, HMQC, and HMBC] indicated the presence of a common 3,5-disubstituted indole moiety in **1** and **2**, and a 1,3,6-trisubstituted tetrahydro-β-carboline moiety in **4**, respectively. In fact, new compounds **1** and **2** differ from **5**–**8** only by the substituents at C-3 of the indole ring, while **4** differs from **3** only by the substituents at C-1 of β-carboline system.

**Table 1:** NMR Data of Compounds **1** *^a^* and **2** *^b^*.

Position	1		2	
	δ_C_ Mult	δ_H_ (*J* in Hz)	δ_C_ Mult	δ_H_ (*J* in Hz)
1	-	-	-	-
2	136.0, CH	8.19, s	125.6, CH	7.05, s
3	114.8, qC	-	105.3, qC	-
3a	129.1, qC	-	130.8, qC	-
4	107.8, CH	7.65, d (2.4)	104.0, CH	6.58, d (2.4)
5	155.0, qC	-	150.0, qC	-
6	114.6, CH	6.76, dd (8.4, 2.4)	111.7, CH	6.65, dd (8.4, 2.4)
7	113.9, CH	7.26, d (8.4)	111.6, CH	7.14, d (8.4)
7a	133.1, qC	-	126.9, qC	-
8	197.5, qC	-	138.6, qC	-
9	72.4, CH	5.18, dd (8.7, 4.5)	151.6, CH	6.80, q (7.3)
10	50.4, CH_2_	2.84, dd (16.8, 8.7)	16.5, CH_3_	1.94, d (7.3)
	-	2.95, dd (16.8, 4.5)	-	-
11	209.7, qC	-	194.9, CH	9.52, s
12	31.3, CH_3_	2.22, s	-	-

*^a^* NMR data were measured in CD_3_OD on a Bruker DRX 400 MHz spectrometer; chemical shifts (ppm) are referenced to CD_3_OD (δ 3.33 ppm, 49.5 ppm). Proton coupling constants (*J*) in Hz are given in parentheses. The assignments were based on DEPT, ^1^H-^1^H COSY, HMQC, and HMBC experiments; *^b^* NMR data were measured in CDCl_3_/CD_3_OD (1:1) on a Bruker DRX 400 MHz spectrometer; chemical shifts (ppm) are referenced to CD_3_OD (3.25 ppm, 48.9 ppm). Proton coupling constants (*J*) in Hz are given in parentheses.

**Figure 1:**
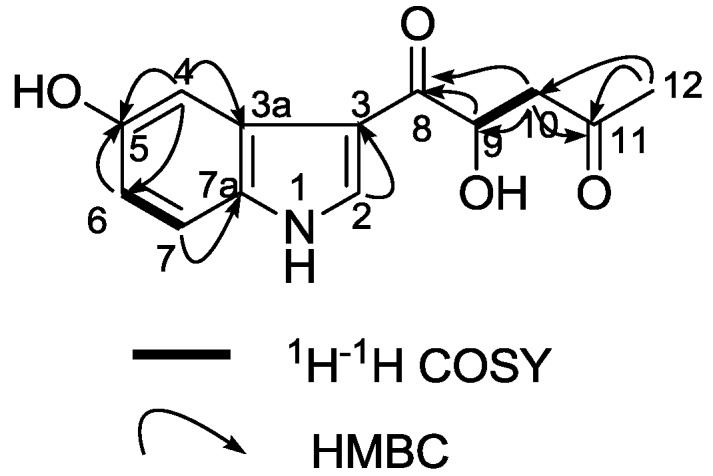
Key 2D NMR correlations of compound **1**.

Hainanerectamine A (**1**) was obtained as an optically active brown amorphous solid (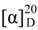 + 4.0 (*c* 0.05, MeOH)). Its negative ESIMS spectrum displayed a pseudo-molecular ion peak at *m/z* 246 [M − H]^−^. The HREIMS experiment established the molecular formula as C_13_H_13_NO_4_ (*m*/*z* 247.0835 [M]^+^, calcd 247.0844), indicating eight degrees of unsaturation. The ^1^H NMR spectrum of **1** showed downfield signals at δ 8.19 (1H, s, H-2), 7.65 (1H, d, H-4), 7.26 (1H, d, H-7), and 6.76 (1H, dd, H-6) characteristic for a 3,5-disubstituted indole moiety [25]. The ^13^C NMR spectrum (Table 1) displayed resonances for 13 carbons including six quaternary carbons, five methines, one methylene and one methyl. Substraction of the above elaborated 5-hydroxyl indole moiety from the molecular formula of **1** indicated that the rest part of the molecule (R_2_), C_5_H_7_O_2_, is composed of two ketons, one oxymethine, one methylene and one methyl. The partial structure 4-hydroxyl-5-oxo-pentan-2-one moiety was readily deduced by the ^1^H-^1^H COSY correlations between H-9 and H_2_-10 and the strong HMBC correlations of H_2_-10/C-8/C-9 and H_3_-12/C-10/C-11 (Figure 1), and was further recognized by comparison of the ^1^H-NMR data of side chain of **1** with those of model compound **9** previously isolated from the sponge *Dysidea etberia* and *Ulosa ruetzler* in racemate form [26]. Finally, the location of R_2_ at C-3 of the indole ring was deduced from the extremely downfield δ value of H-2 (δ 8.19) that implied the presence of a strongly deshielding substituent at C-3. Thus, the planar gross structure of **1** was determined. There is only one chiral center (C-9) in the molecule of **1**. To determine its absolute configuration, the modified Mosher’s method [27,28] was applied since hainanerectamine A contains a secondary hydroxyl group at C-9. Thus, compound **1** was esterified separately with (*R*)- and (*S*)-MTPA chloride in dry pyridine at room temperature. Unfortunately, we failed to obtain the expected corresponding MTPA esters of **1** due to the decomposition of the molecule during the reaction**.** Whereas limited amounts of **1** prevent us to try again the Mosher’s reaction. The configuration of hydroxyl group at C-9 of **1** thus remains undefined.

**Figure 2:**
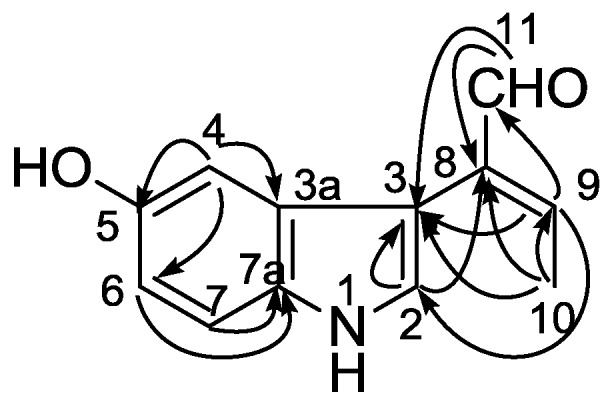
Key HMBC correlations of compound **2**.

Hainanerectamine B (**2**), a brown amorphous solid, showed the molecular ion peak at *m/z* 201 [M]^+^ in the EIMS, and the molecular formula, C_12_H_11_NO_2_, was established by HREIMS (*m/z* 201.0787, calcd 201.0790). The IR spectrum showed the absorptions indicated the presence of OH and/or NH (3390 cm^−1^) group(s) and conjugated aldehyde (2925 and 1629 cm^−1^). The ^1^H and ^13^C NMR spectra of **2** clearly showed the presence of an aldehyde group (δ_H_ 9.52, 1H, s; δ_C_ 194.9, d) and suggested the presence of the same 3,5-disubstituted indole nucleus as **1**. To account for the molecular formula, the side chain (R_2_) at C-3 has to be a butenal group (C_4_H_5_O). Furthermore, the splitting pattern of H-9 (6.80, q, *J* = 7.3 Hz), H_3_-10 (1.94, d, *J* = 7.3 Hz) and H-11 (9.52, s) suggested that the olefin at ∆^8^ is conjugated with the aldehyde. The distinct NOE cross peak between H-9 and H-11 indicated the *E* nature of the double bond. Finally, a series of long-range C-H correlations (Figure 2) between H-2 (δ 7.05, s)/C-3 (δ 105.3) and C-8 (δ 138.6), H-4 (δ 6.58, d, *J* = 2.4 Hz)/C-3a (δ 130.8), C-5 (δ 150.0) and C-6 (δ 111.6), H-9/C-2 (δ 125.6), C-3 and C-11 (δ 194.9), H_3_-10/C-3, C-8 and C-9 (δ 151.6), H-11/C-3 and C-8 clearly confirmed the position of the 2-butenal and hydroxyl groups to be on C-3 and C-5, respectively. Thus, the structure of compound **2** was unambiguously assigned.

Both compounds **3** and **4** were isolated from the *n*-BuOH fraction implying that they are more polar than co-occurring indole alkaloids. Moreover, **3** and **4** showed other common spectroscopic properties: they appeared as strong greenish fluorescence spots on TLC plates under UV light (λ = 366 nm); their UV spectra displayed strong absorption bands at λ_max_ 275 nm (log ε 3.4) and 358 nm (log ε 2.7); their IR spectra indicated the presence of OH and/or NH (3405 cm^−1^) and carbonyl (1628 cm^−1^) groups.

**Figure 3:**
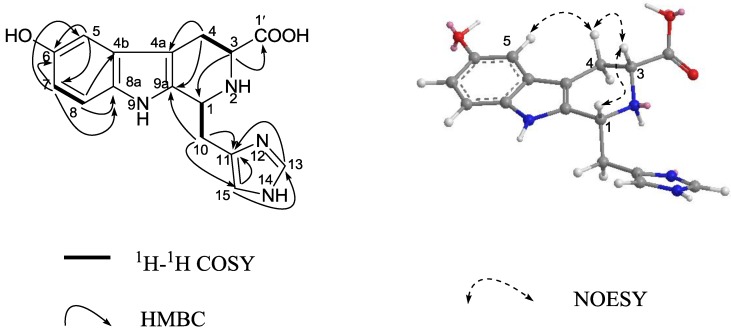
Key 2D NMR correlations of compound **3**.

Compound **3** was purified as a yellow solid. Its positive HRESIMS displayed a molecular ion peak at *m/z* 313.1301, indicating a molecular formula of C_16_H_17_N_4_O_3_ for the [M + H]^+^ and suggesting 11 degrees of unsaturation. The ^1^H NMR spectrum of **3** displayed five downfield multiplets between δ 7.7 and δ 6.7, and six aliphatic protons between δ 4.8 and δ 2.9. The ^13^C NMR spectrum showed resonances for 16 carbons including seven methines, two methylenes, and seven quaternary carbons (Table 2). Interpretation of the ^1^H, ^13^C, ^1^H-^1^H COSY, HMQC, and HMBC NMR spectra led to the assembly of three spin systems within **3**. The first spin coupling system is an ABC system including signals at δ 6.82 (d, H-5), 6.68 (dd, H-7), and 7.17 (d, H-8). The second spin system comprised H-3β/4α (*J* = 13.6 Hz), H-3β/4β (*J* = 4.0 Hz), H-4α/4β (*J* = 15.3 Hz). The remaining coupling system included H-1 (δ 4.80, m), H_2_-10 [(δ 3.48, m) and (δ 3.18, m)], and two imidazole signals corresponding to H-13 (δ 7.68) and H-15 (δ 7.00). The placement of the methylimidazole and carboxylic acid moieties at C-1 and C-3, respectively, was supported by the HMBC experiments, which showed H_2_-10/C-9a (δ 131.6), C-11 (δ 136.0) and C-15 (δ 117.2) and H-3 (δ 3.86, m)/C-1′ (δ 174.9) connectivities. Similarly, the assignment of all quaternary carbons within **3** was secured from HMBC correlations (Figure 3). The NOE correlations of H-5/H-4 (δ 2.90), H-4/H-3, and H-3/H-1 clearly indicated that the carboxylic acid and the imidazole side chain were oriented on the same face. From the above evidences, the structure of compound **3** was determined as (−)-(1R^*^, 3R^*^)-6-hydroxy-1-(4-methylene-1*H*-imidazole)-2,3,4,9-tetrahydro-1*H*-β-carboline-3-carboxylic acid. 

**Table 2:** NMR Data of Compounds **3** and **4** *^a^*.

Position	3		3 *^b^*		4	
	δ_C_ Mult	δ_H_ (*J* in Hz)	δ_C_ Mult	δ_H_ (*J* in Hz)	δ_C_ Mult	δ_H_ (*J* in Hz)
1	55.8, CH	4.80, m	54.6	5.01, br, s	55.2, CH	4.72, m
2	-	-	-	-	-	-
3	60.2, CH	3.86, m	59.7	4.26, br, d (7.3)	60.6, CH	3.70, m
4	25.0, CH_2_	3.30, dd (15.3, 4.0)	24.2	3.36, m	25.7, CH_2_	3.28, dd (15.4, 3.8)
	-	2.90, dd (15.3, 13.6)	-	3.04, dd (15.1, 12.4)	-	2.96, dd (15.4, 13.8)
4a	108.5, qC	-	107.9	-	109.3, qC	-
4b	128.7, qC	-	128	-	129.1, qC	-
5	103.8, CH	6.82, d (2.1)	103.4	6.86, d (2.3)	103.8, CH	6.84, d (2.4)
6	152.4, qC	-	152.2	-	152.4, qH	-
7	113.7, CH	6.68, dd (8.7, 2.1)	113.8	6.75, dd (8.7, 2.3)	113.5, CH	6.67, dd (8.7, 2.4)
8	113.3, CH	7.17, d (8.7)	113.1	7.22, d (8.7)	113.2, CH	7.17, d (8.7)
8a	133.7, qC	-	133.4	-	133.8, qC	-
9	-	-	-	-	-	-
9a	131.6, qC	-	129.7	-	130.7, qC	-
10	31.0, CH_2_	3.48, m	28.8	3.73, br, d (14.2)	28.4, CH_2_	3.64, m
	-	3.18, m	-	3.36, m	-	3.24, m
11	136.0, qC	-	130.7	-	130.7, qC	-
12	-	-	-	-	-	-
13	137.7, CH	7.68, s	136.2	8.73, s	141.0, CH	7.69, s
14	-	-	-	-	-	-
15	117.2, CH	7.00, s	118.6	7.27, s	134.6, qC	-
COOH	174.9, qC	-	170.6	-	176.0, qC	-
NCH_3_	-	-	-	-	33.3, CH_3_	3.71, s
SCH_3_	-	-	-	-	19.8, CH_3_	2.39, s

*^a^* NMR data were measured in CD_3_OD on a Bruker DRX 400 MHz spectrometer; chemical shifts (ppm) are referenced to CD_3_OD (δ 3.33 ppm, 49.5 ppm). Proton coupling constants (*J*) in Hz are given in parentheses. The assignments were based on DEPT, ^1^H-^1^H COSY, HMQC, and HMBC experiments; *^b^* NMR data were reported in the literature [29]. Chemical shifts (ppm) are referenced to CD_3_OD (δ 3.30 ppm, 49.0 ppm).

Literature survey revealed that the structure of alkaloid **3** was the same as that of hyrtioreticulin B, a metabolite isolated previously from the sponge *H. reticulates* by Tsukamoto *et al.* [29]. However, careful comparison of the overall ^1^H- and ^13^C-NMR dataof compound **3** with those of hyrtioreticulin B (Table 2) revealed significant differences between them. For instance, the ∆δ_H_ of chemical shift for H-13 in both compounds was more than 1.0 ppm and the differences of the ^13^C-NMR data were even more obvious, especially for the C-11 (∆δ_c_ = +4.8 ppm) and -COOH (∆δ_c_ = +3.8 ppm). From a natural product chemistry point of view, the differences are too big to be ignored. In order to secure the correctness of **3**, the 1D and 2D NMR spectra were reanalyzed and the proposed structure was confirmed. It raises a necessity to clarify if the isolate **3** and hyrtioreticulin B are the same. Surprisingly, the ^1^H- and ^13^C-NMR spectra of hyrtioreticulin B are quite different from those of **3**. We failed to obtain authentic sample of hyrtioreticulin B from Tsukamoto’s group for the direct comparison. At this stage, we can only ascribe these differences to the “alkaloidal nature” [30]. 

**Figure 4:**
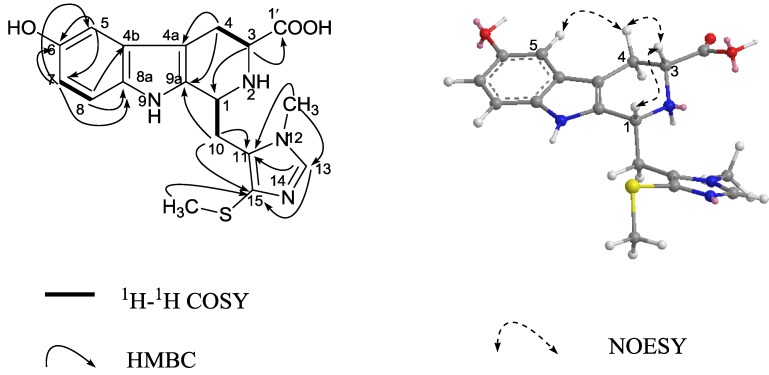
Key 2D NMR correlations of compound **4**.

Compound **4** was obtained as a yellow solid. The ESIMS spectrum of **4** showed a pseudo-molecular ion peak at *m/z* 373 [M + H]^+^, and its molecular formula was established as C_18_H_21_N_4_O_3_S from the HRESIMS (*m*/*z* 373.1348 ([M + H]^+^, calcd 373.1334), indicating 11 degrees of unsaturation. The ^1^H and ^13^C NMR (Table 2) spectra of **4** showed great similarities to those of **3**. The presence of a same 3- carboxylic acid, 6-hydroxyl functional groups on the tetrahydro-β-carboline ring as **3** was immediately recognized. In addition, NMR spectra displayed additional signals for one N-methyl group [δ_H_ 3.71 (3H, s), δ_C_ 33.3 (CH_3_)] and one thiomethyl group [δ_H_ 2.39 (3H, s), δ_C_ 19.8 (CH_3_)], that should be obviously substituted on the imidazole ring in agreement with the observed molecular weight difference of 60 mass units. Significant ^1^H-^13^C long-range correlation, as show in Figure 4, allowed to locate the methyl to N-12 and the thiomethyl group to C-15 and connected the imidazole system at C-10. Thus, the planar structure of **4** was unambiguously assigned. By analogy to Compound **3**, the relative configuration of H-1 and H-3 in **4** was assigned as *cis* by the ROESY experiment (Figure 4). On the basis of above observations, the structure of **4** was determined to be 12-N-CH_3_, 15-SCH_3_ derivative of **3**, named hainanerectamine C.

In conclusion, besides the sesterterpenoids that were already reported previously, six indole alkaloids and two β-carboline derivatives were also isolated and structurally characterized from the present collection (LS-103) of Hainan sponge *H. erecta*. It is interesting to note that although many indole alkaloids were reported from various natural resources [31], to the best of our knowledge, this is the first report of the isolation of indole alkaloid bearing a butenal moiety from a marine sponge. On the other hand, although many β-carboline alkaloids were reported from the genus *Hyrtios* and other marine sponges [32], only two tetrahydro-β-carboline containing both a methylimidazole and a carboxylic acid moiety at C-1 and C-3 have been reported from the animals of this genus [29]. The abundant production and accumulation of indole and β-carboline alkaloids in the title specimen is intriguing, as it seems unlikely that these compounds act solely as repellents against predators. Instead, they may play as an as yet unknown physiological role in these benthic animals.

Compound **1**–**8** were evaluated for their inhibitory activity against Aurora A [33,34,35]. Bioassay results showed that compounds **2**–**4** had moderate inhibitory effects with IC_50_ values of 24.5, 13.6, and 18.6 μg/mL, respectively. The cytotoxic activities of **1**–**8** against the growth of several tumor cell lines [A549 (human lung adenocarcinoma) and HT-29 (human promyelocytic leukemia)] were also evaluated. Unfortunately, the results indicated that all the tested compounds were inactive against the above cancer cells (50% effective dose of clonal inhibition ED_50_ > 10 μg/mL).

## 3. Experimental Section

### 3.1. General Experimental Procedures

Optical rotations were measured on a Perkin-Elmer 241MC polarimeter. UV spectra were recorded on a Varian Cary 300 Bio spectrophotometer. IR spectra were recorded on a Nicolet-Magna FT-IR 750 spectrometer. NMR spectra were recorded on a Bruker DRX-400 spectrometer (Bruker, Germany) with the residual CH_3_OH (δ_H_ 3.30 ppm, δ_C_ 49.0 ppm) as an internal standard. EIMS and HREIMS data were obtained on a Finnigan-MAT-95 mass spectrometer. ESIMS and HRESIMS spectra were recorded on a Waters Q-TOF Micro LC-MS-MS mass spectrometer. Reversed-phase HPLC (Agilent 1100 series liquid chromatography using a VWD G1314A detector at 210 nm and a semi-preparative ODS-HG-5 (5 μm, 10 mm (i.d.) × 25 cm) column) was also employed. Commercial Si gel (Qing Dao Hai Yang Chemical Group Co., Qingdao, China, 200–300 and 400–600 mesh) was used for CC, and precoated Si gel plates (Yan Tai Zi Fu Chemical Group Co., Yantai, China, G60 F-254) were used for analytical TLC. The Aurora kinase inhibitor VX-680 was from Roche (purity = 99.88%). Z-LYTE Kinase Assay Kit (Cat No. PV3174, Invitrogen, Carlsbad, CA, USA).

### 3.2. Animal Material

The specimens of *H. erecta* were collected off Lingshui Bay, Hainan Province, China, in November 2003, and were identified by Prof. Rob W. M. van Soest (Zoologisch Museum, University of Amsterdam, Amsterdam, The Netherlands). A voucher sample (LS-103) is available for inspection at the Herbarium of Shanghai Institute of Materia Medica, CAS, Shanghai, China. 

### 3.3. Extraction and Separation

The frozen sponge (190 g, dry weight) was extracted with acetone (1 L × 4) exhaustively at room temperature. The acetone extract was concentrated *in vacuo* and the resulting residue (5.6 g) was partitioned between Et_2_O and H_2_O, *n*-BuOH and H_2_O, respectively. The Et_2_O-soluble extract (2.6 g) was chromatographed on a silica gel column (5 × 40 cm) using light petroleum ether with increasing amount of EtOAc (10:0, 9:1, 8:2, 7:3, 6:4, 5:5, 4:6, 3:7, 0:10, v/v, each 1L) as eluent. The fraction eluted with EtOAc/petroleum ether (4:6, 415.6 mg) was further purified by Silica gel CC (3 × 30 cm) eluting with CHCl_3_/MeOH (9:1) to give the pure compounds **1** (>95%, 5.7 mg) and **2** (>95%, 8.2 mg). The *n*-BuOH-soluble extract (>95%, 2.5 g) was subjected to gel filtration on Sephadex LH-20 (CH_3_OH, 4 × 110 cm), yielding a mixture that was further purified by RP-HPLC [semipreparative ODS-HG-5 (5 μm, 250 × 10 mm), CH_3_OH/H_2_O (4:6), 2.0 mL/min] to afford two pure compounds **3** (>95%, 6.2 mg) and **4** (>95%, 5.1 mg).

Compound **1**: brown amorphous solid; 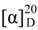 + 4.0 (*c* 0.05, MeOH); UV (MeOH) λ_max_ (log ε) 353 (0.7), 307 (3.4), 252 (3.7), 271 (3.6), 213 (4.0) nm; IR (KBr) ν_max_ 3405, 2929, 1705, 1629, 1473, 1076 cm^−1^; EIMS *m/z* 247 [M]^+^ (10), 160 (100), 132 (22); HREIMS *m/z* 247.0835, calcd for C_13_H_13_NO_4_, 247.0844; ^1^H and ^13^C NMR see Table 1.

Compound **2**: gray amorphous powder; UV (MeOH) λ_max_ (log ε) 357 (3.1), 356 (3.0), 276 (3.8), 254 (3.6), 219 (4.5) nm; IR (KBr) ν_max_ 3396, 2925, 2854, 1672, 1630, 1461, 1166 cm^−1^; EIMS *m/z* 201 [M]^+^ (100), 172 (68), 139 (56), 127 (24), 111 (28); HREIMS *m/z* 201.0787, calcd for C_12_H_11_NO_2_, 201.0784; ^1^H and ^13^C NMR data see Table 1.

Compound **3**: yellow solid; 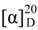 −47 (*c* 0.06, MeOH); UV (MeOH) λ_max_ (log ε) 358 (2.7), 286 (3.3), 275 (3.4), 205 (3.9) nm; IR (KBr) ν_max_ 3405, 2927, 1626, 1400, 1201, 1084, 625 cm^−1^; ESIMS *m/z* 313 [M + H]^+^; HRESIMS *m/z* 313.1301, calcd for C_16_H_17_N_4_O_3_, 313.1301; ^1^H and ^13^C NMR data see Table 2.

Compound **4**: yellow solid; 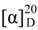 −40 (*c* 0.06, MeOH); UV (MeOH) λ_max_ (log ε) 389 (2.8), 358 (2.9), 302 (3.3), 271 (3.5), 204 (4.0) nm; IR (KBr) ν_max_ 3423, 1627, 1402, 1201, 1143, 1035, 621 cm^−1^; ESIMS *m/z* 373 [M + H]^+^; HRESIMS *m/z* 373.1348, calcd for C_18_H_21_N_4_O_3_S, 373.1334; ^1^H and ^13^C NMR see Table 2. 

### 3.4. Biological Assay

Compounds **1**–**8** were evaluated for their inhibitory activity against Aurora A, a member of serine/threonine kinase family involving in the regulation of cell division and a new target in cancer treatment [33,34,35]. Compounds **1**–**8** were initially diluted to a 100× concentration in DMSO. The 100× concentrations were then diluted to a 4× working concentration in kinase buffer. 2.5 μL of the test compounds solution and the positive control at various concentrations were added to a barcoded corning, low volume NBS, 384-well plate, then 5 μL of the 2× peptide/kinase mixture and 2.5 μL of the 4× ATP solution were added to the plate. The assay plate was shaken on a plate shaker for 30 s, and then incubated for 60 min at room temperature. After the treatment 5 μL of the development reagent solution was added to the plate. The assay plate was shaken on a plate shaker for 30 s, and then incubated for 60 min at room temperature. The assay plate is read on fluorescence plate reader and the data is analyzed.

## 4. Conclusions

Our investigation demonstrated that the sponge *H. erecta* is a good source of bioactive substances. Compounds **2**–**4** exhibited moderate inhibitory activity against Aurora A, a member of serine/threonine kinase family involving in the regulation of cell division and a new target in cancer treatment, with IC_50_ values of 24.5, 13.6, and 18.6 μg/mL, respectively. These results suggest that continuing investigation of novel secondary metabolites together with the potentially useful bioactivities from this marine organism are worthwhile for future drug development. Moreover, further study should be conducted to unambiguously confirm the identity of structures of compound **3** and hyrtioreticulin B by total synthesis in future.

## Supplementary Files

Supplementary File 1 Supplementary Information (PDF, 4409 KB)

## Supplementary Information

NMR and HR-ESI-MS spectra of the new compounds are available in Supplementary Information.
