# Supplementary material for: Hainanerectamines A–C, Alkaloids from the Hainan Sponge Hyrtios erecta
Source: Mar Drugs. 2014 Jun 30;12(7):3982–93. doi: 10.3390/md12073982 (PMC4113810; doi:10.3390/md12073982)
Supplement: Supplementary File 1 — Supplementary Information (PDF, 4409 KB) [file marinedrugs-12-03982-s001.pdf]

Supplementary Information

Figure S1. ^{1}H NMR spectrum of Hainanerectamine A.

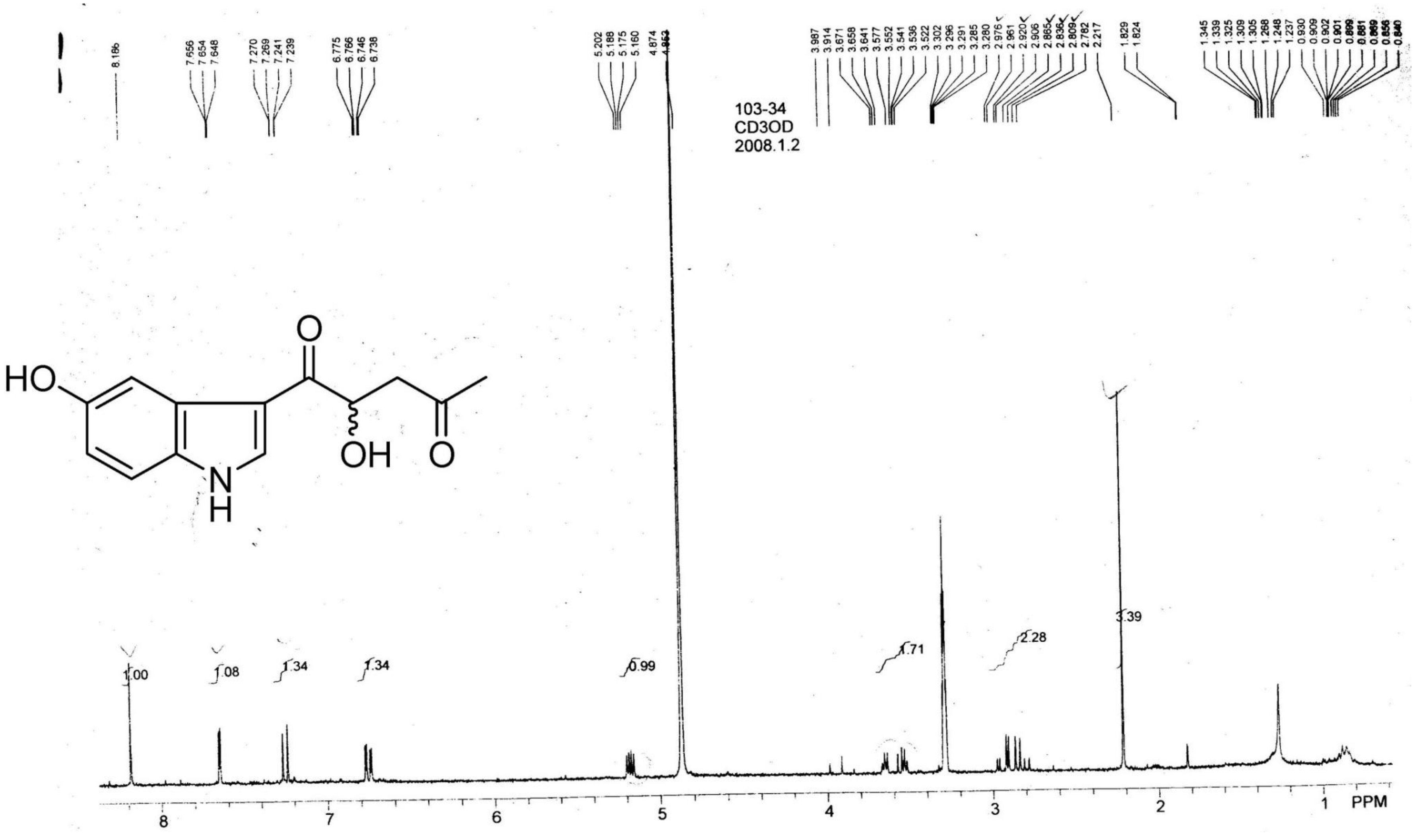

Figure S2. ^{13}C NMR spectrum of Hainanerectamine A.

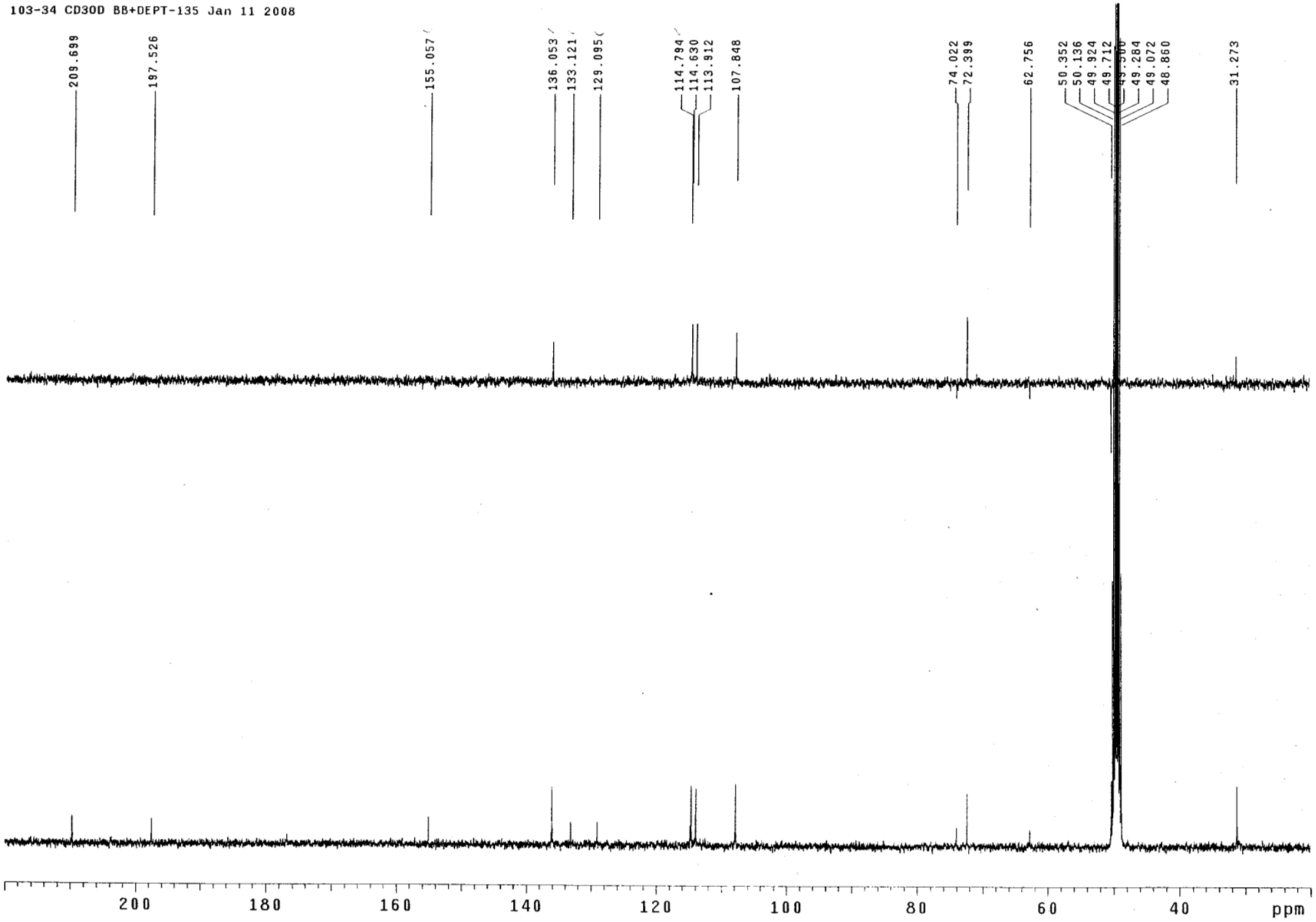

Figure S3. HSQC spectrum of Hainanerectamine A.

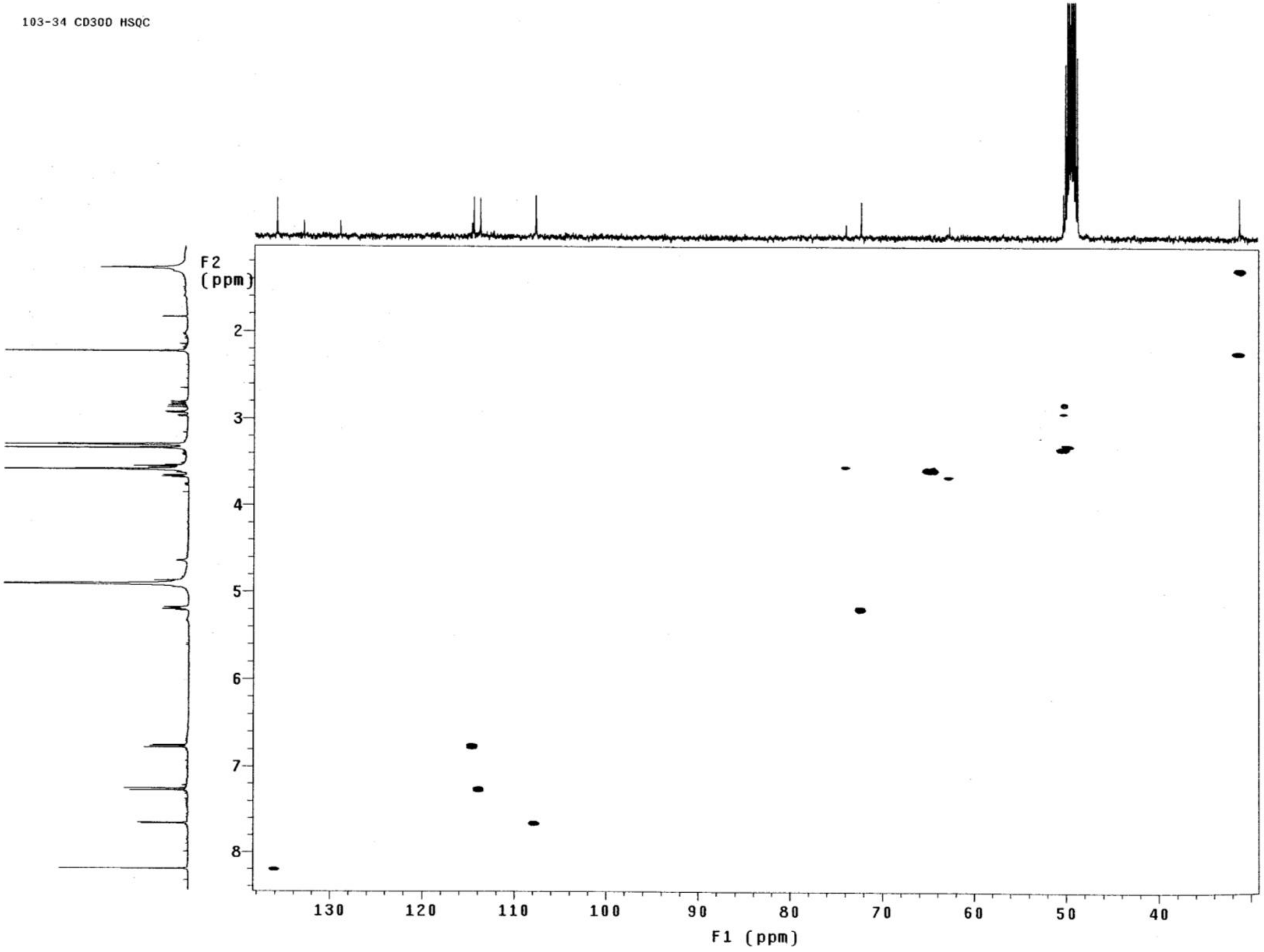

Figure S4. HMBC spectrum of Hainanerectamine A.

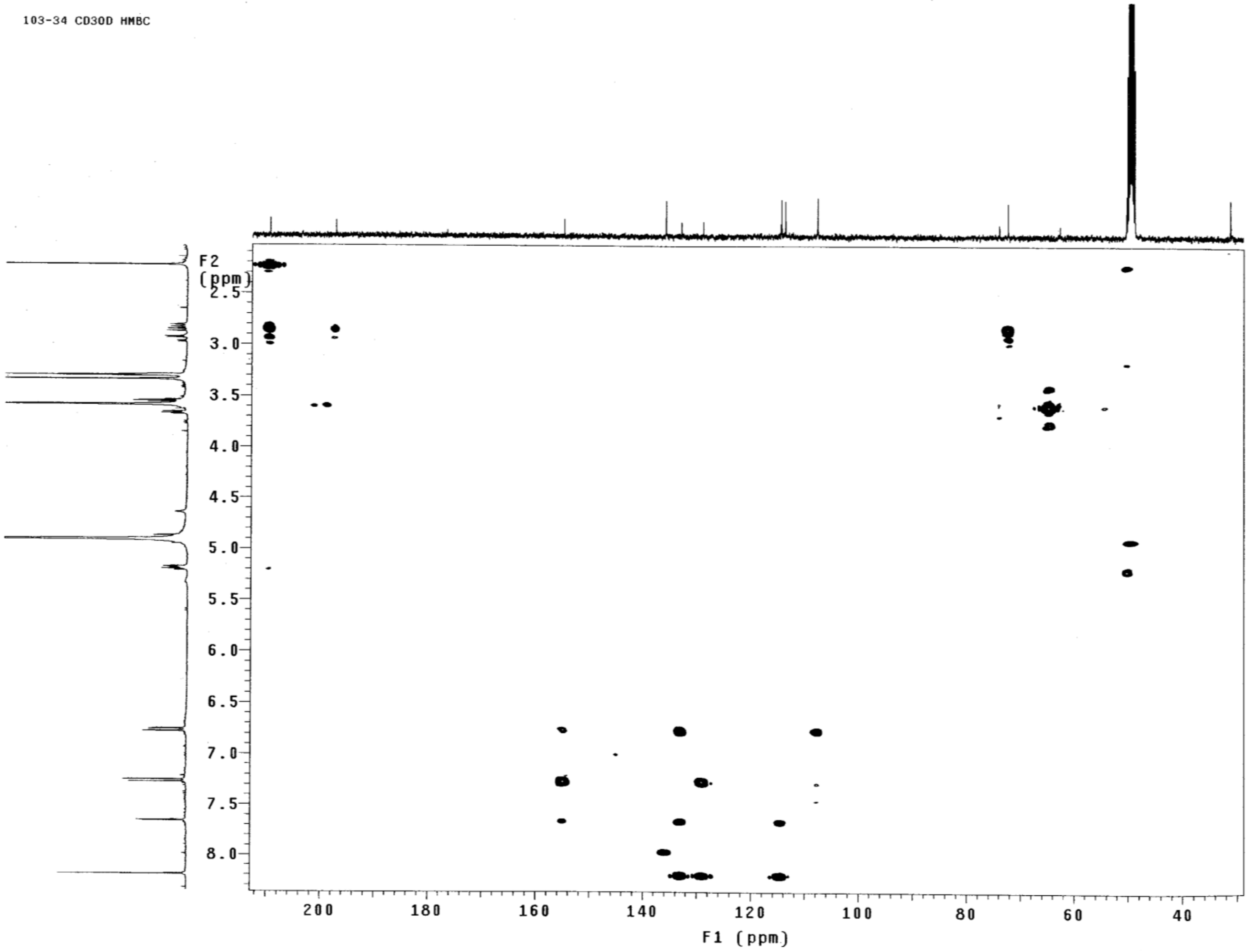

Figure S5. ^{1}H ^{1}H COSY spectrum of Hainanerectamine A.

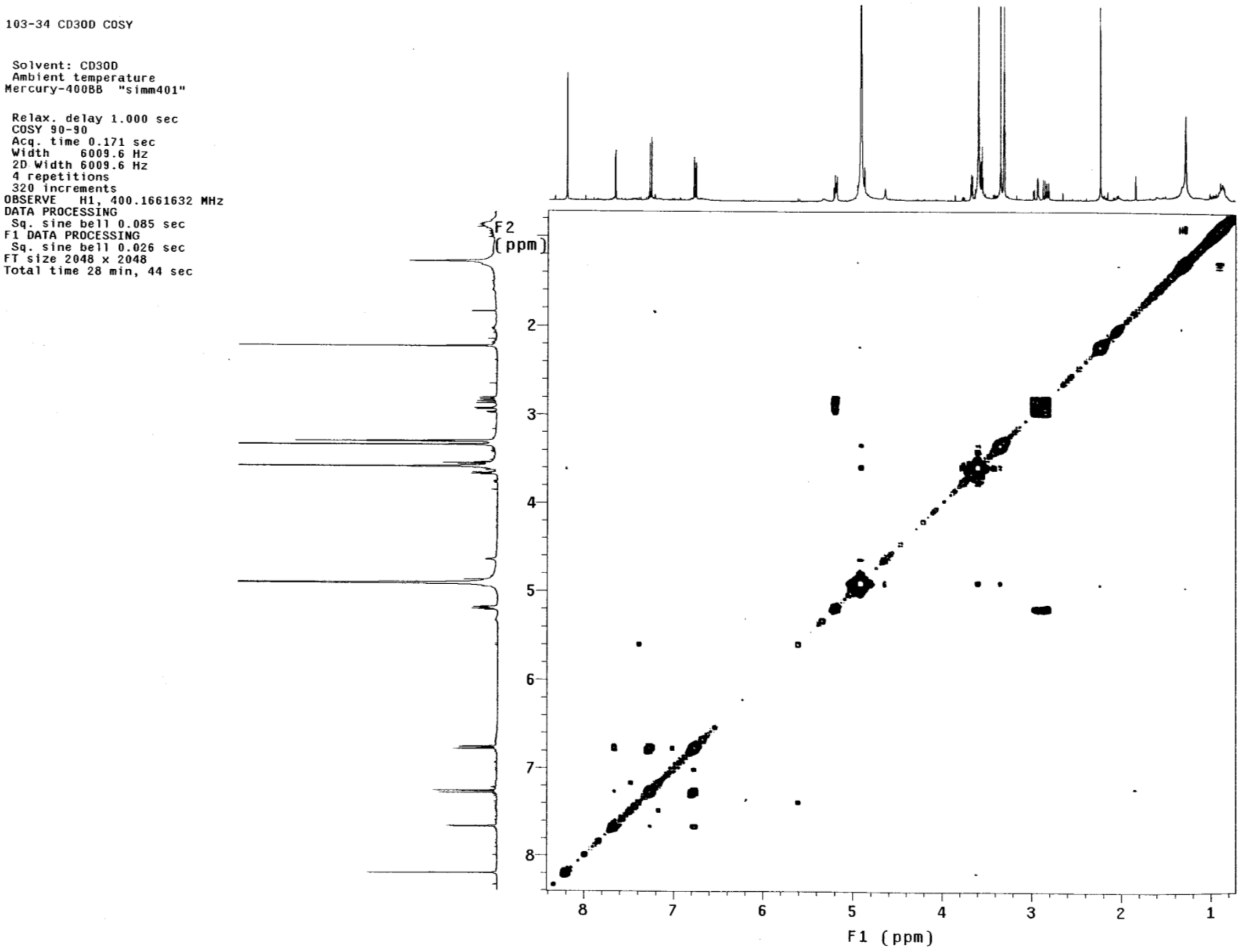

Figure S6. HREI spectrum of Hainanerectamine A.

```
LIST:  h80151-c1                           18-Jan-08   Elapse:   05:22.4       25
Samp:  103-34                                          Start :   14:04:12      27
Comm:  Finnigan/MAT95//70eV/Tsou:200c/R:10000
Mode:  EI +VE +LMR  BSCAN (EXP) UP HR NRM
Oper:  WANG_J@SIMM.CAS   Client:  S/N:SW01-0001        Inlet :
Limt: (   0 )     .    . .
    : ( 347 ) C14.H100.N.O4
Peak:  1000.00  mmu    R+D:  -2.0 > 60.0
Data:  CMASS : converted
```

Mass		Intensity (4705)	%RA	%RIC	Delta (mmu)	R+D	Composition
51.02992	*	4939	1.05	0.02	2.1	-1.0	H5.N.O2
76.03226	*	4760	1.01	0.02	-1.0	5.0	C6.H4
77.03959	*	9283	1.97	0.04	-0.5	4.5	C6.H5
103.0400	*	6545	1.39	0.03	-0.5	1.5	C4.H7.O3
					2.2	6.0	C7.H5.N
105.0319	*	18090	3.84	0.08	2.2	5.5	C7.H5.O
131.0386	*	6069	1.29	0.03	-1.5	7.0	C8.H5.N.O
132.0455	*	48498	10.31	0.22	-0.5	6.5	C8.H6.N.O
133.0510	*	7438	1.58	0.03	-0.9	1.5	C5.H9.O4
					1.8	6.0	C8.H7.N.O
146.9877	*	7021	1.49	0.03			
159.0318	*	10889	2.31	0.05	0.3	8.0	C9.H5.N.O2
160.0392	*	470587	100.00	2.15	0.6	7.5	C9.H6.N.O2
161.0425	*	50284	10.69	0.23			
162.9932	*	6783	1.44	0.03	-2.7	8.0	C7.H.N.O4
173.9850	*	31658	6.73	0.14	-2.3	9.5	C8.N.O4
186.0546	*	9461	2.01	0.04	0.9	8.5	C11.H8.N.O2
196.9839	*	4998	1.06	0.02			
229.0736	*	16721	3.55	0.08	0.3	9.0	C13.H11.N.O3
247.0835	*	46237	9.83	0.21	0.9	8.0	C13.H13.N.O4

Figure S7. ^{1}H NMR spectrum of Hainanerectamine B.

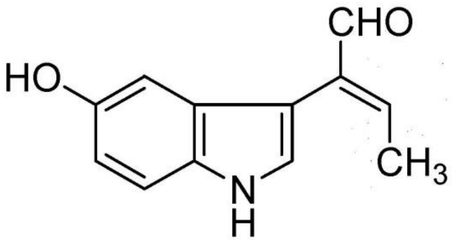

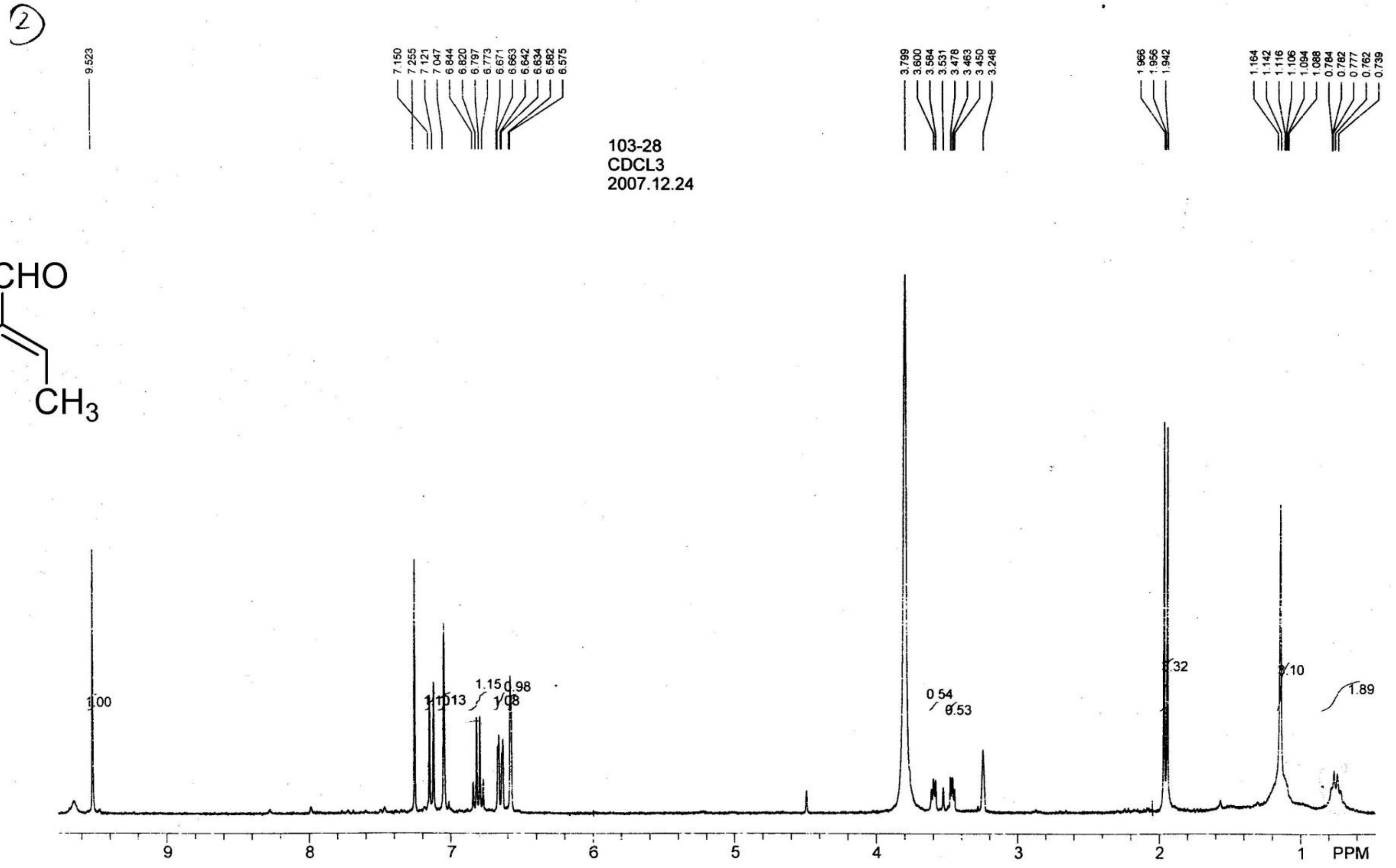

Figure S8. ^{13}C NMR spectrum of Hainanerectamine B.

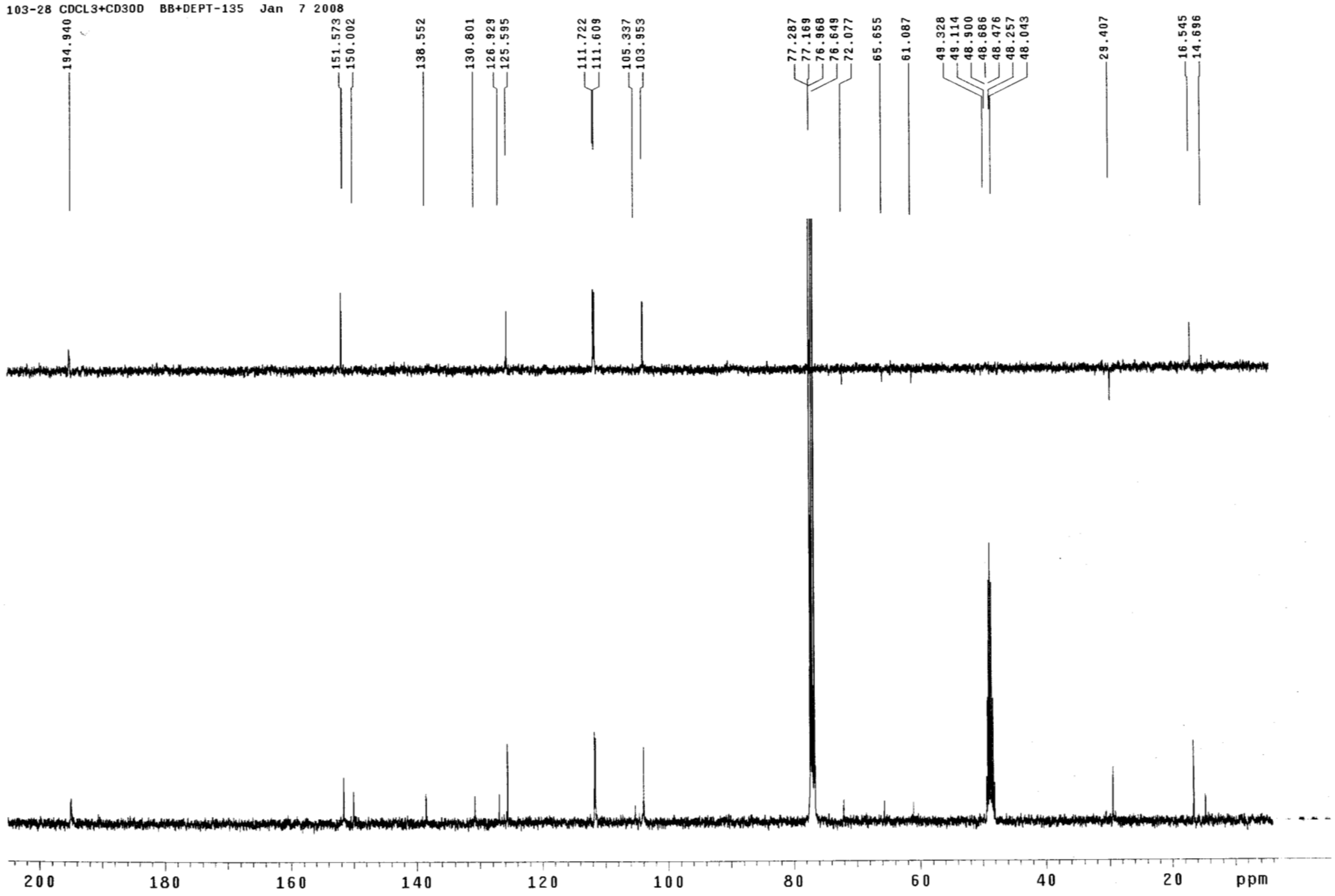

Figure S9. HSQC spectrum of Hainanerectamine B.

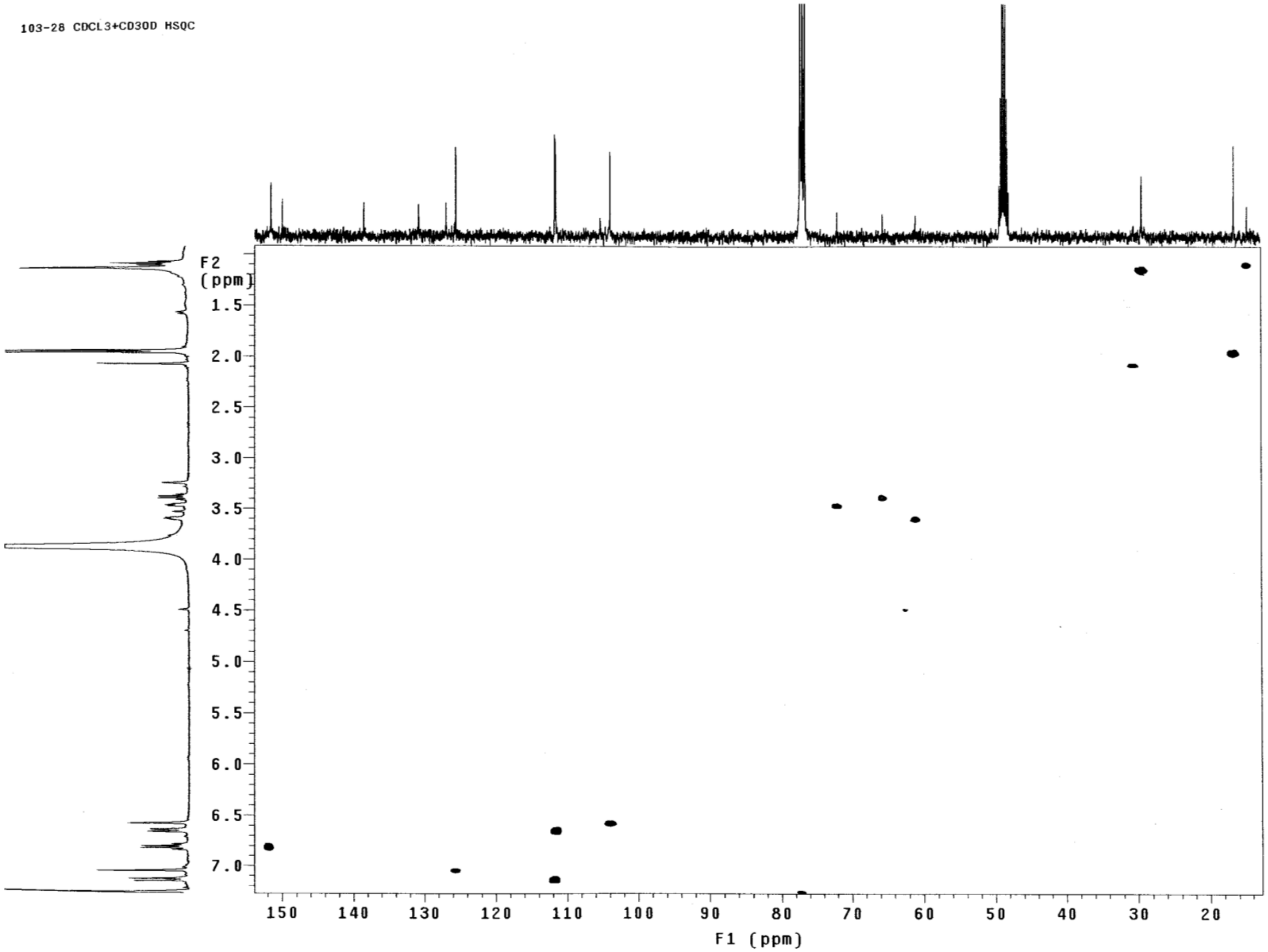

Figure S10. HMBC spectrum of Hainanerectamine B.

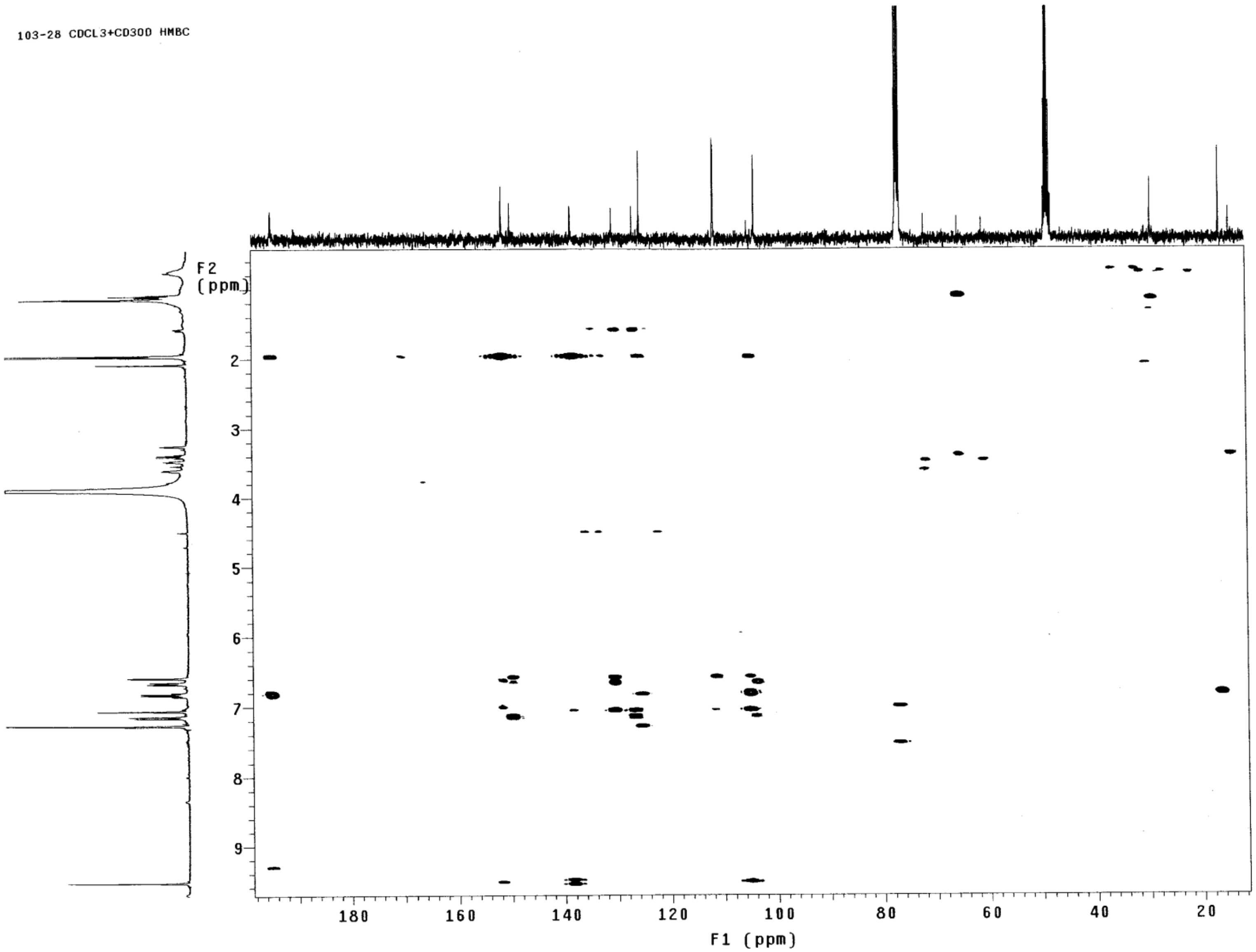

Figure S11. NOE spectrum of Hainanerectamine B.

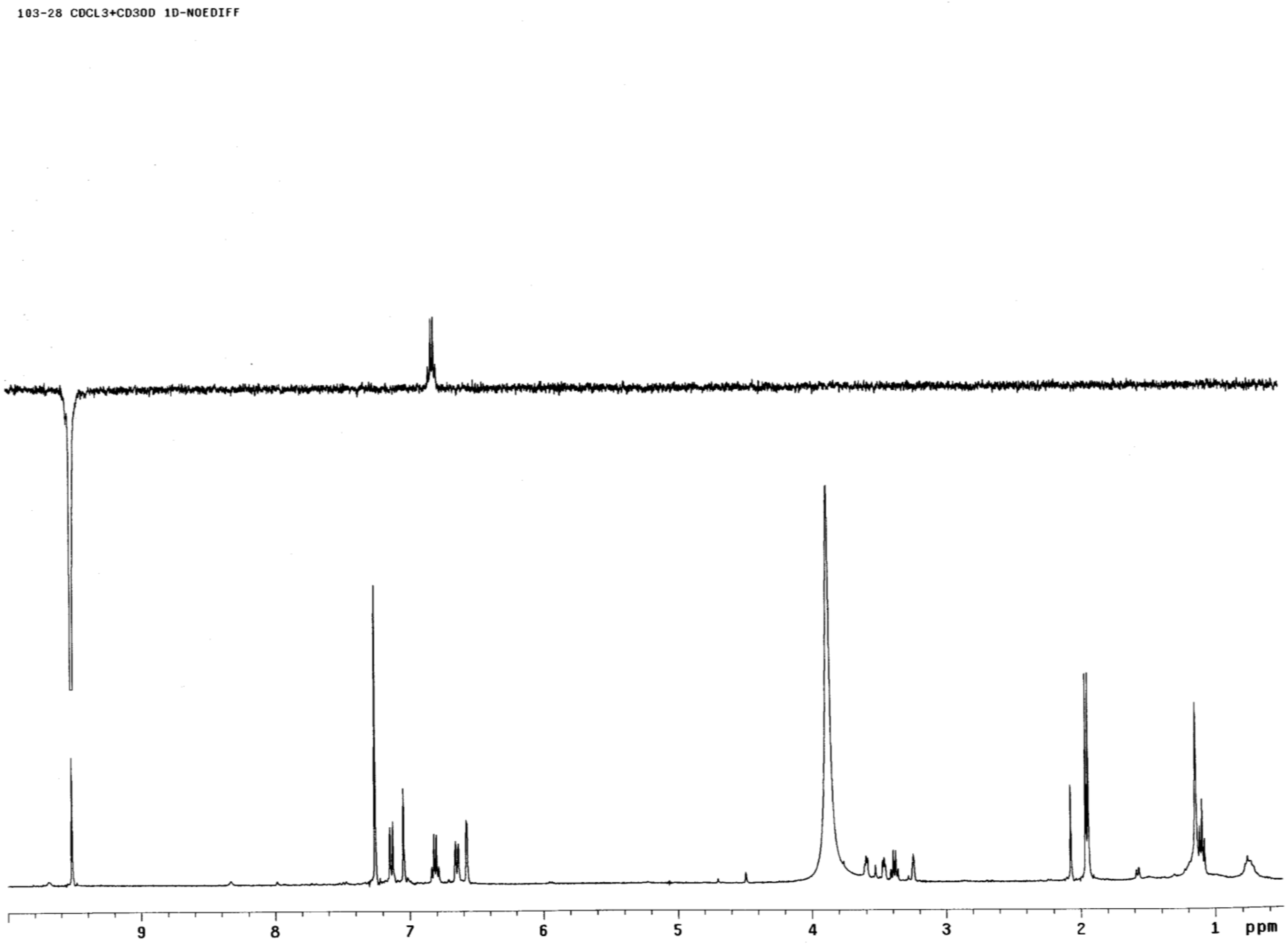

Figure S12. HREI spectrum of Hainanerectamine B.

```
LIST:  h80150-c1                              18-Jan-08   Elapse:  08:31.5       41
Samp:  103-28                                             Start :  13:52:28      43
Comm:  Finnigan/MAT95//70eV/Tsou:200c/R:10000
Mode:  EI +VE +LMR  BSCAN (EXP) UP HR NRM
Oper:  WANG_J@SIMM.CAS   Client:  S/N:SW01-0001           Inlet :
Limt: (    0 )       .     . .
    : ( 303 ) C13.H100.N.O2
Peak:  1000.00  mmu    R+D:   -2.0 > 60.0
Data:  CMASS : converted
```

	5628			(mmu)		
Mass	Intensity	%RA	%RIC	Delta	R+D	Composition
51.0307 *	10949	3.89	0.04	1.3	-1.0	H5.N.O2
55.0639 *	8509	3.02	0.03			
57.0785 *	13091	4.65	0.04			
59.0457 *	10175	3.62	0.03			
63.0330 *	15353	5.46	0.05	-1.0	0.0	C.H5.N.O2
65.0486 *	8985	3.19	0.03	-0.9	-1.0	C.H7.N.O2
69.0705 *	7616	2.71	0.02	-0.1	1.5	C5.H9
71.0864 *	7259	2.58	0.02	-0.3	0.5	C5.H11
73.0309 *	6902	2.45	0.02	-2.0	1.5	C3.H5.O2
77.0398 *	9699	3.45	0.03	-0.7	4.5	C6.H5
89.0380 *	11782	4.19	0.04	1.1	5.5	C7.H5
91.0542 *	11068	3.93	0.04	0.5	4.5	C7.H7
102.0485 *	6486	2.30	0.02	-1.6	6.0	C8.H6
108.9938 *	7974	2.83	0.03			
115.0573 *	38799	13.79	0.13	-2.6	6.5	C9.H7
116.0538 *	9402	3.34	0.03			
117.0588 *	7200	2.56	0.02	-1.0	6.0	C8.H7.N
117.0712 *	12080	4.29	0.04	-0.8	5.5	C9.H9
127.0547 *	39334	13.98	0.13	0.0	7.5	C10.H7
128.0499 *	6069	2.16	0.02	0.1	7.5	C9.H6.N
133.0523 *	13091	4.65	0.04	0.4	6.0	C8.H7.N.O
142.0652 *	12080	4.29	0.04	0.5	7.5	C10.H8.N
143.0733 *	13448	4.78	0.04	0.2	7.0	C10.H9.N
144.0456 *	8807	3.13	0.03	-0.6	7.5	C9.H6.N.O
144.0581 *	8033	2.85	0.03	-0.6	7.0	C10.H8.O
144.0815 *	19994	7.10	0.07	-0.2	6.5	C10.H10.N
145.0514 *	9045	3.21	0.03	1.4	7.0	C9.H7.N.O
145.0643 *	26123	9.28	0.09	1.0	6.5	C10.H9.O
146.0623 *	15174	5.39	0.05	-1.7	6.5	C9.H8.N.O
146.9863 *	10175	3.62	0.03			
149.0245 *	13567	4.82	0.04	2.0	11.0	C11.H3.N
154.0653 *	21541	7.65	0.07	0.3	8.5	C11.H8.N
155.0723 *	6843	2.43	0.02	1.2	8.0	C11.H9.N
156.0806 *	13924	4.95	0.05	0.7	7.5	C11.H10.N
157.0530 *	16840	5.98	0.06	-0.2	8.0	C10.H7.N.O
158.0598 *	13270	4.72	0.04	0.8	7.5	C10.H8.N.O
160.0393 *	16483	5.86	0.05	0.5	7.5	C9.H6.N.O2
170.0604 *	27314	9.71	0.09	0.2	8.5	C11.H8.N.O
171.0665 *	15888	5.65	0.05	2.0	8.0	C11.H9.N.O
172.0752 *	193578	68.79	0.63	1.1	7.5	C11.H10.N.O
173.0811 *	44630	15.86	0.15			
173.9852 *	42250	15.01	0.14			
196.9841 *	8390	2.98	0.03			
199.0630 *	6188	2.20	0.02	0.3	9.0	C12.H9.N.O2
200.0705 *	8152	2.90	0.03	0.7	8.5	C12.H10.N.O2
201.0787 *	281412	100.00	0.92	0.3	8.0	C12.H11.N.O2
202.0820 *	29337	10.42	0.10			

Figure S13. ^{1}H NMR spectrum of Compound **3**.

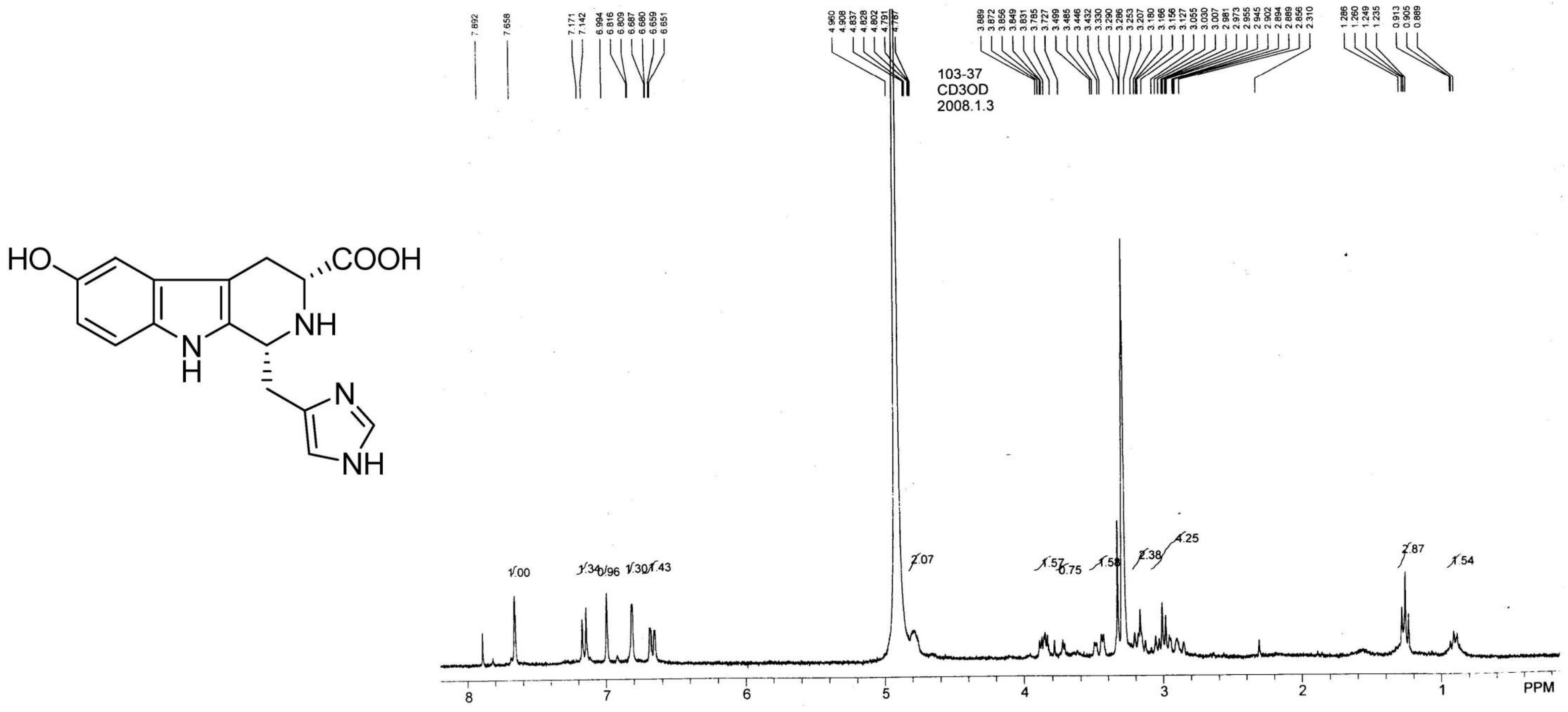

Figure S14. ^{13}C NMR spectrum of Compound **3**.

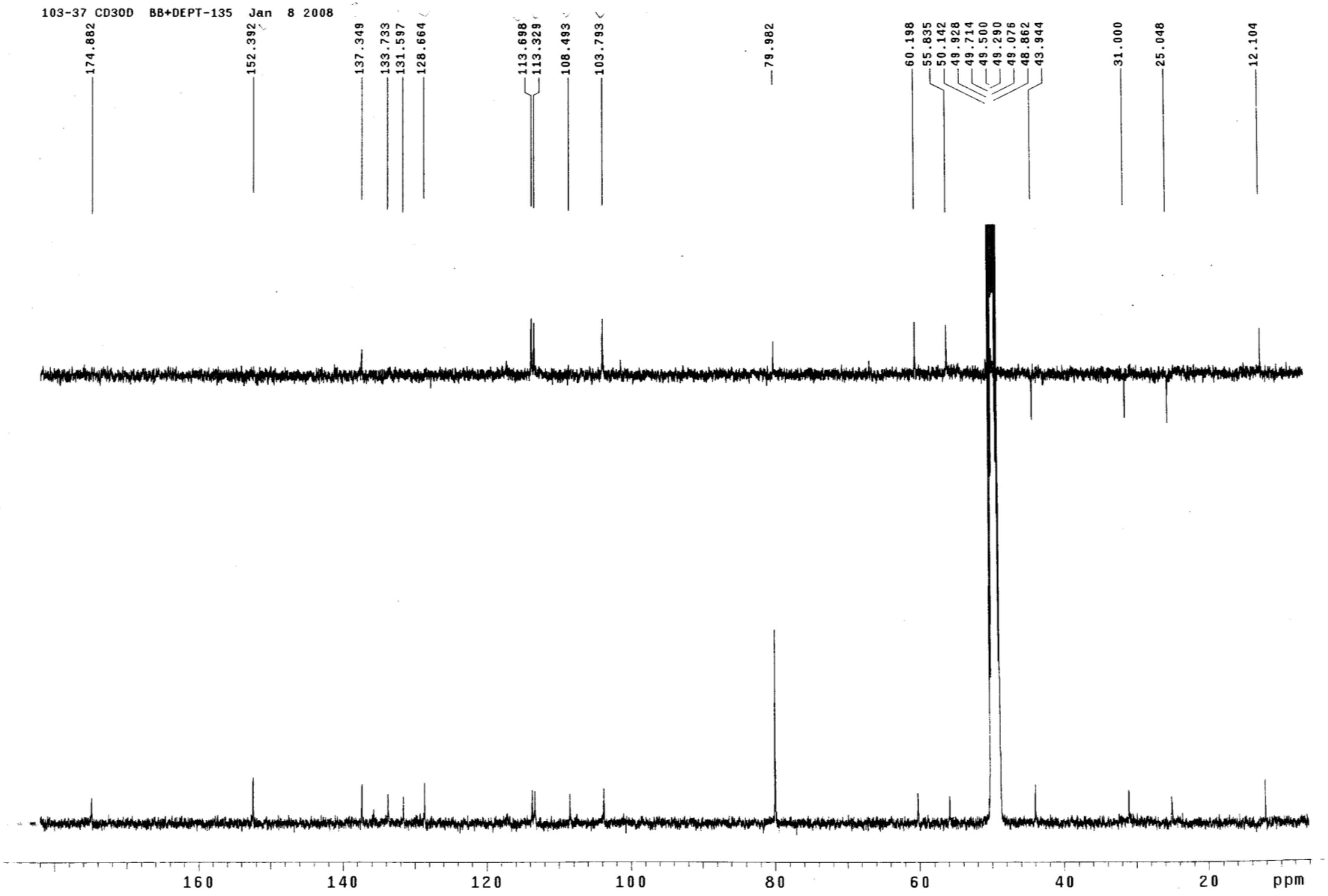

Figure S15. ^1H-^1H COSY spectrum of Compound **3**.

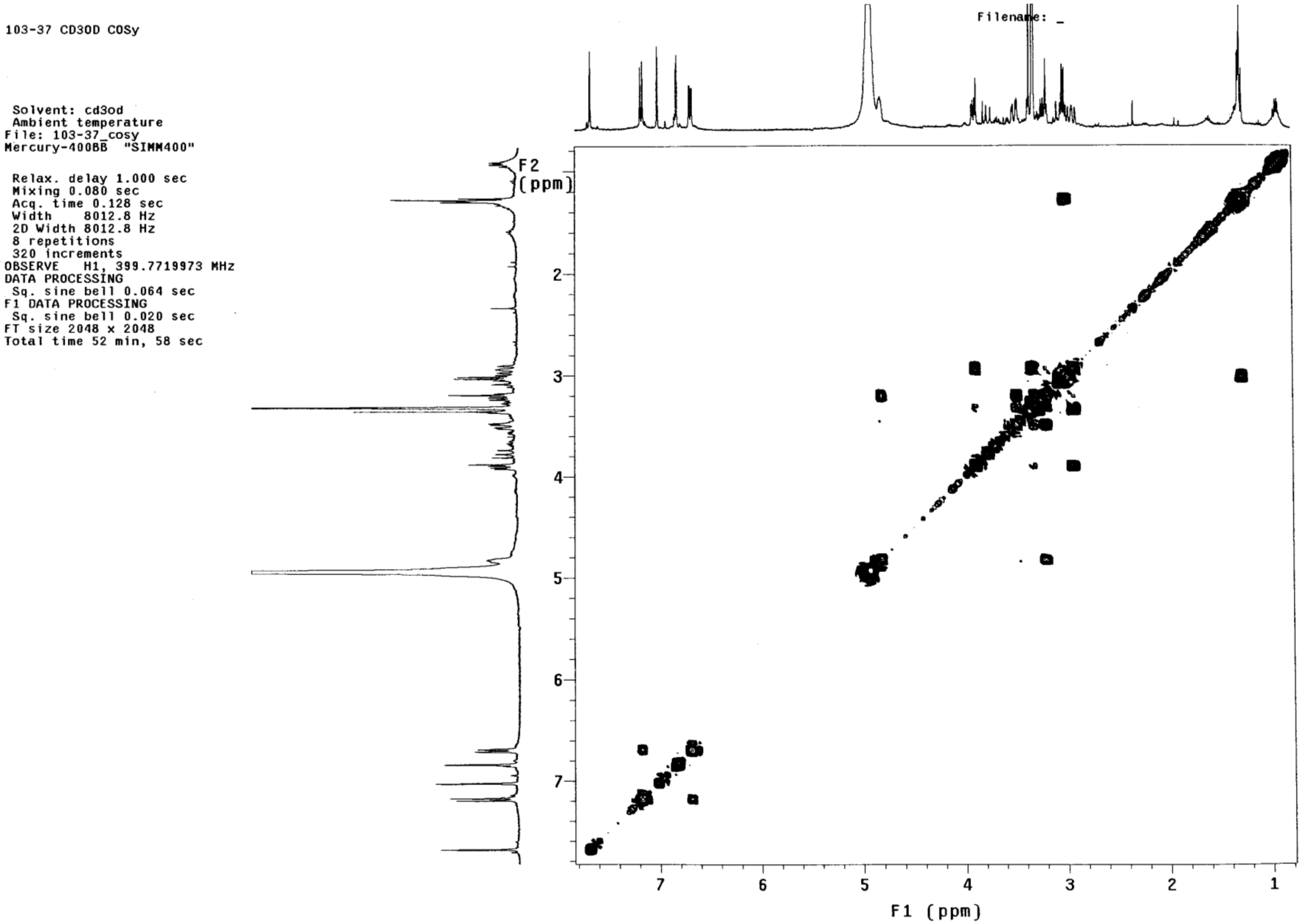

Figure S16. HSQC spectrum of Compound **3**.

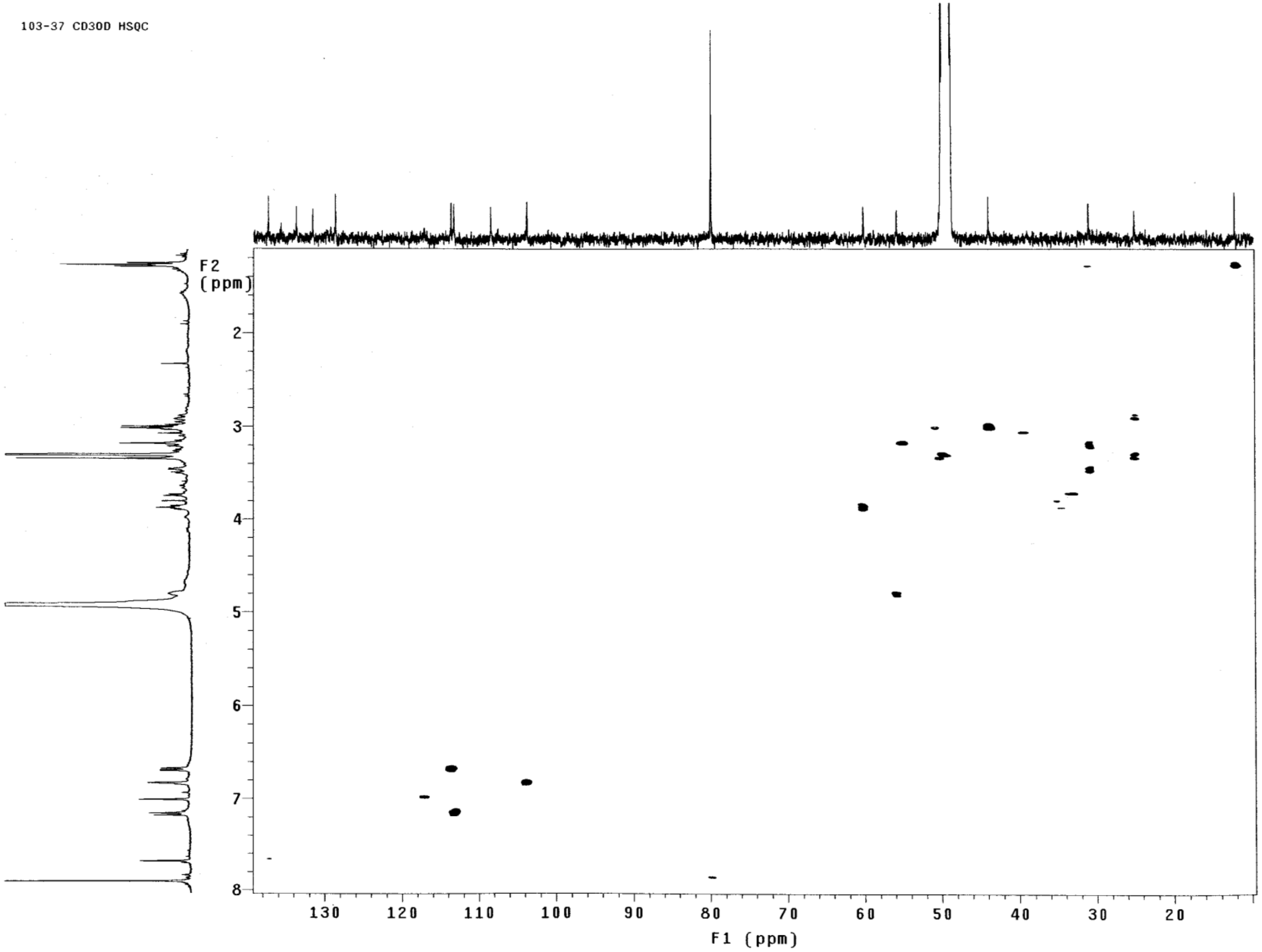

Figure S17. HMBC spectrum of Compound **3**.

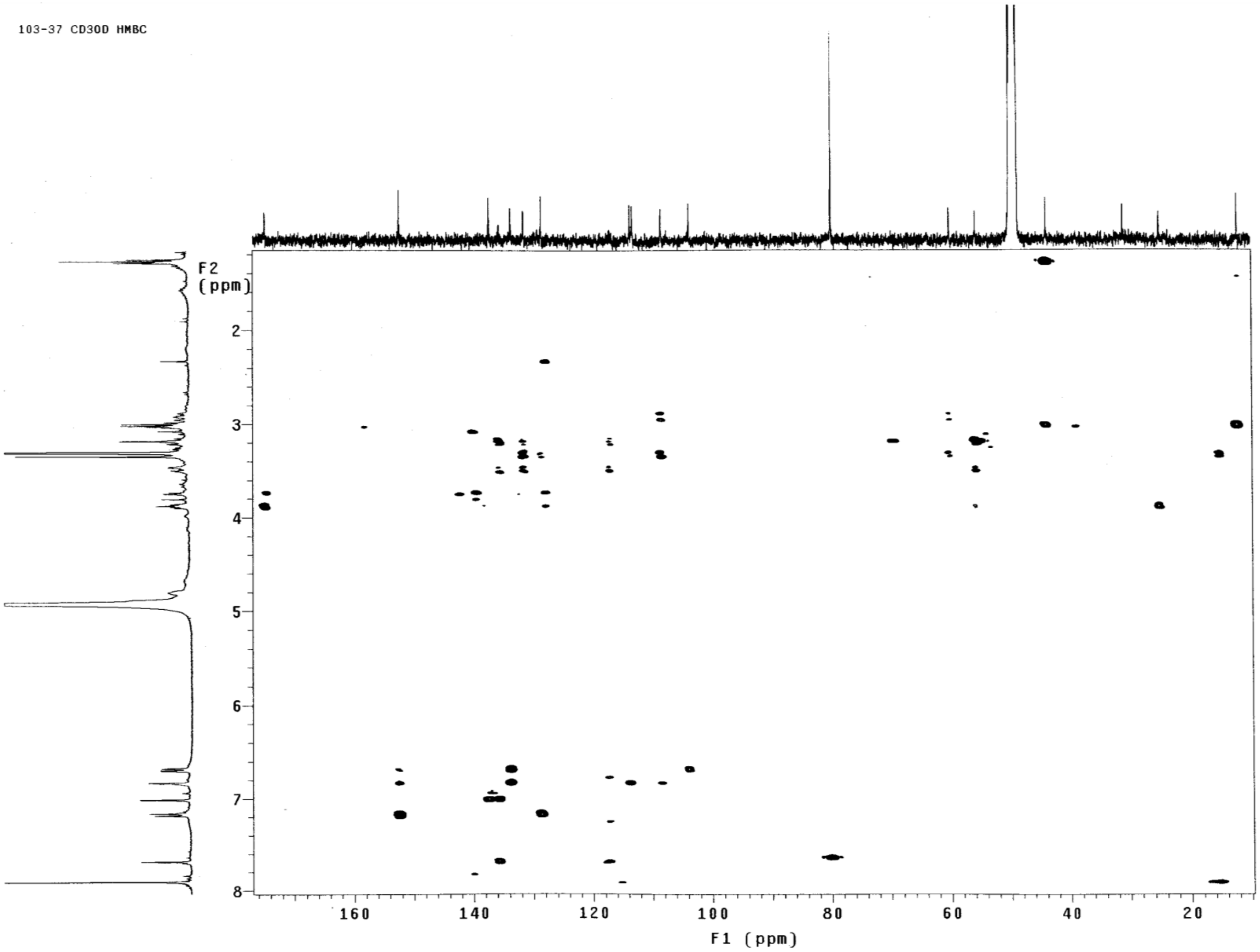

Figure S18. ROESY spectrum of Compound **3**.

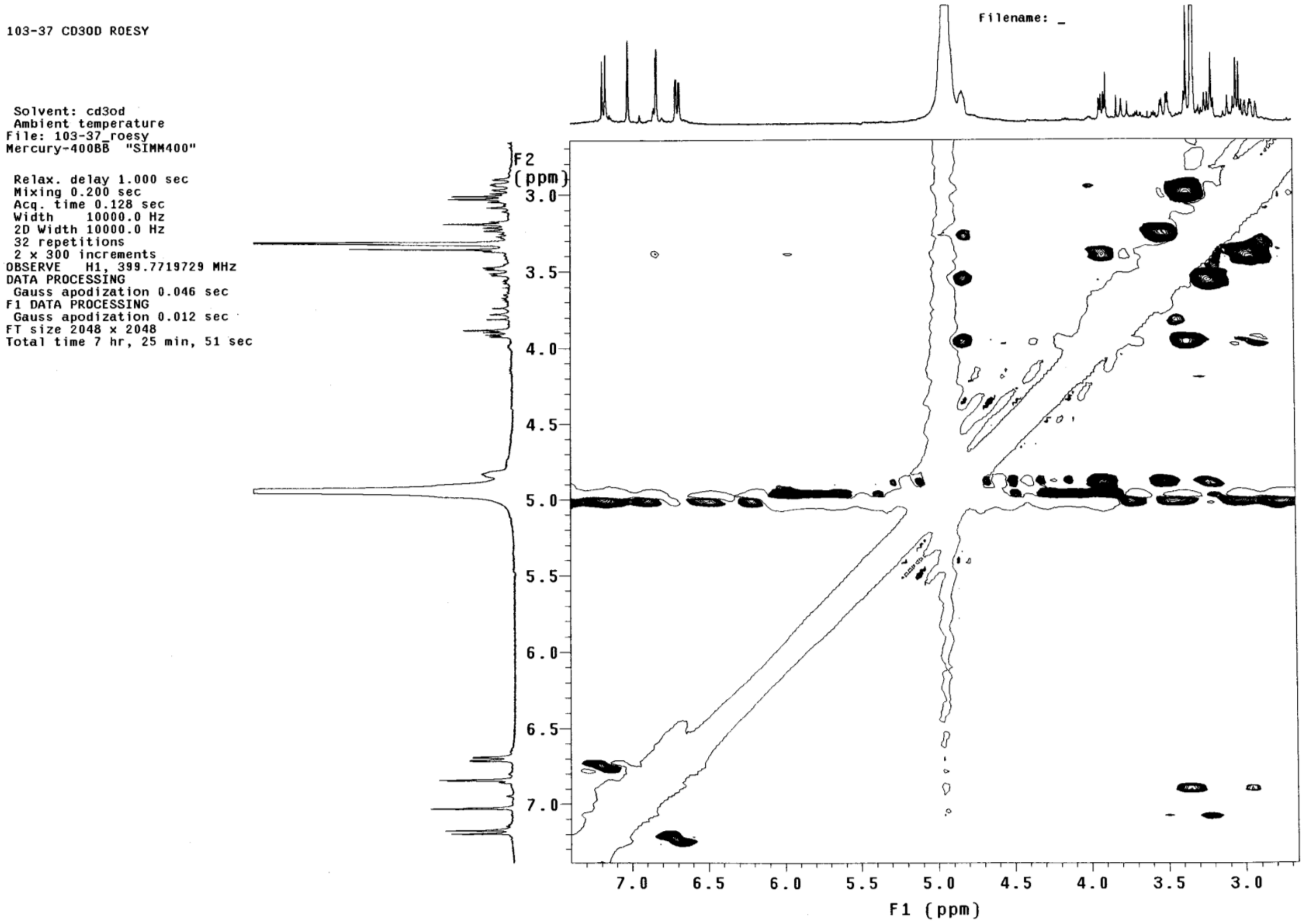

Figure S19. HRESI spectrum of Compound **3**.

Elemental Composition Report

Page 1

Tolerance = 50.0 PPM / DBE: min = -1.5, max = 50.0
Isotope cluster parameters: Separation = 1.0 Abundance = 1.0%

Monoisotopic Mass, Odd and Even Electron Ions
24 formula(e) evaluated with 1 results within limits (up to 20 closest results for each mass)

080222-2 123 (2.316) AM (Cen,2, 40.00, Ht,5000.0,362.93,1.00); Sm (SG, 2x3.00); Cm (117:145)
TOF MS ES+

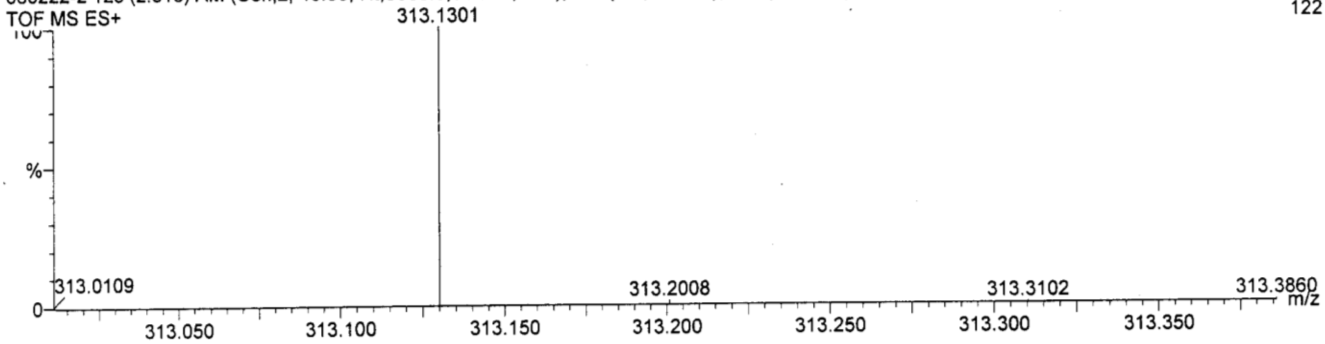

Minimum:	50.00				-1.5		
Maximum:	100.00		200.0	50.0	50.0		

Mass	RA	Calc. Mass	mDa	PPM	DBE	Score	Formula
313.1301	100.00	313.1301	0.0	0.1	10.5	1	C16 H17 N4 O3

Figure S20. IR spectrum of Compound **3**.

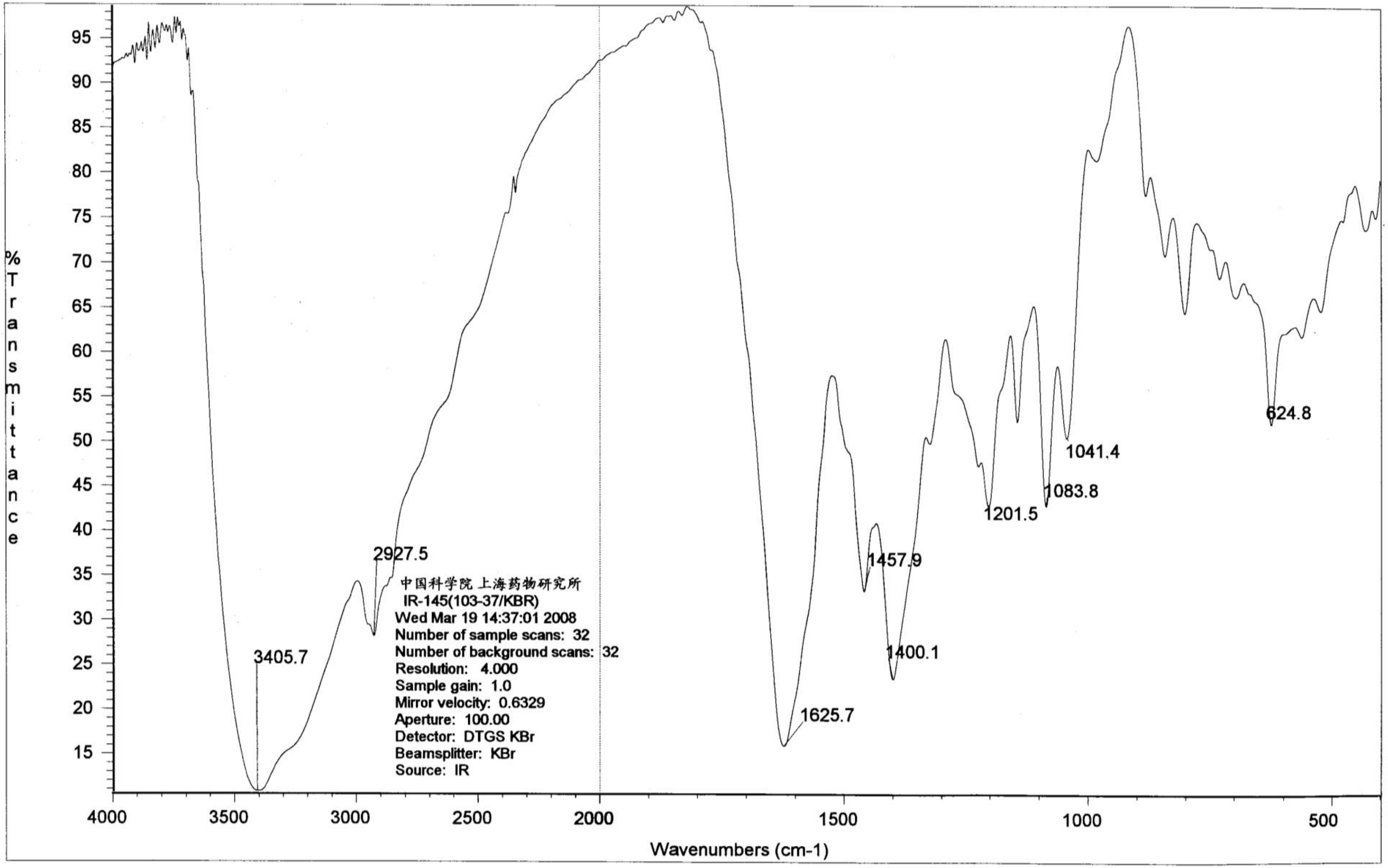

Figure S21. ^{1}H NMR spectrum of Hainanerectamine C.

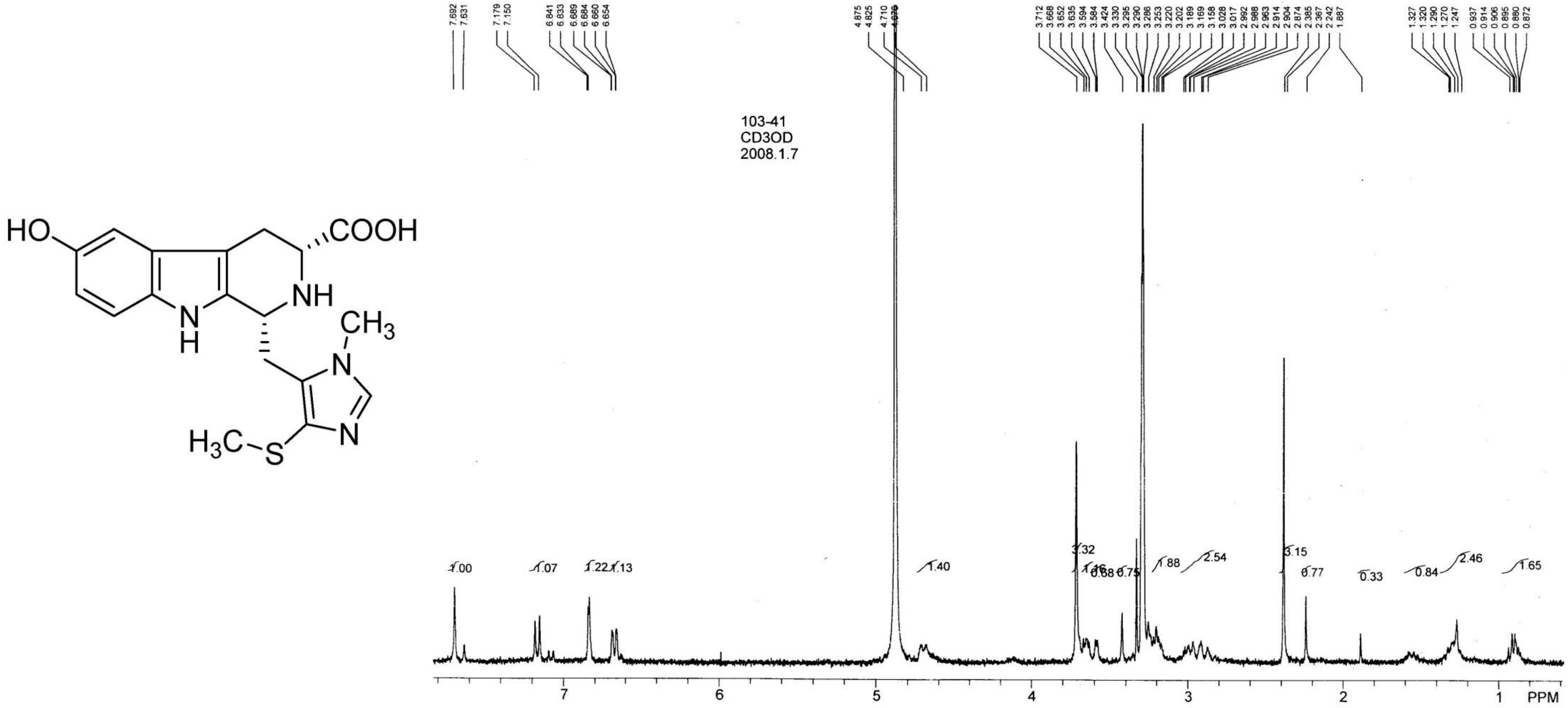

Figure S22. ^{13}C NMR spectrum of Hainanerectamine C.

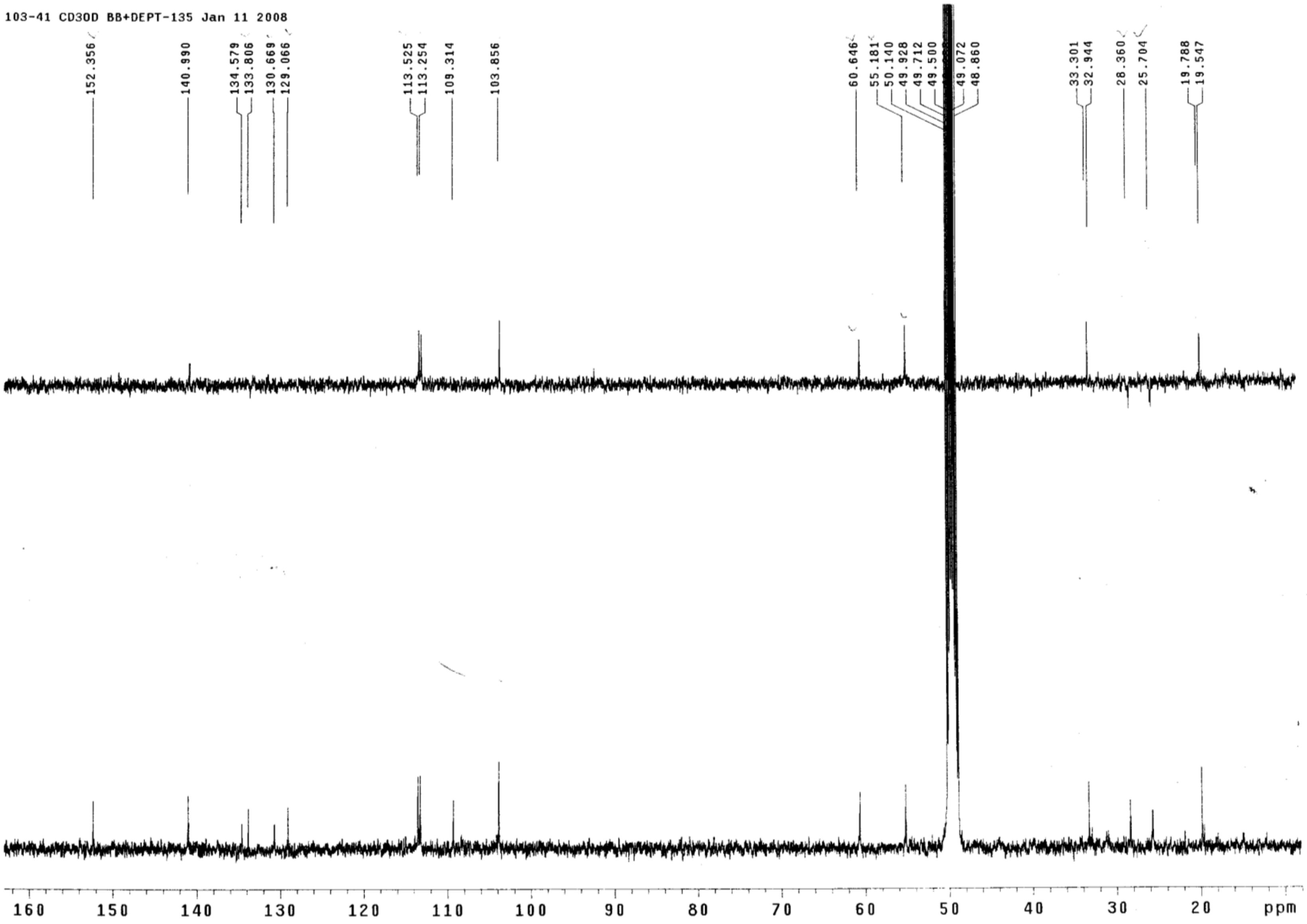

Figure S23. HSQC spectrum of Hainanerectamine C.

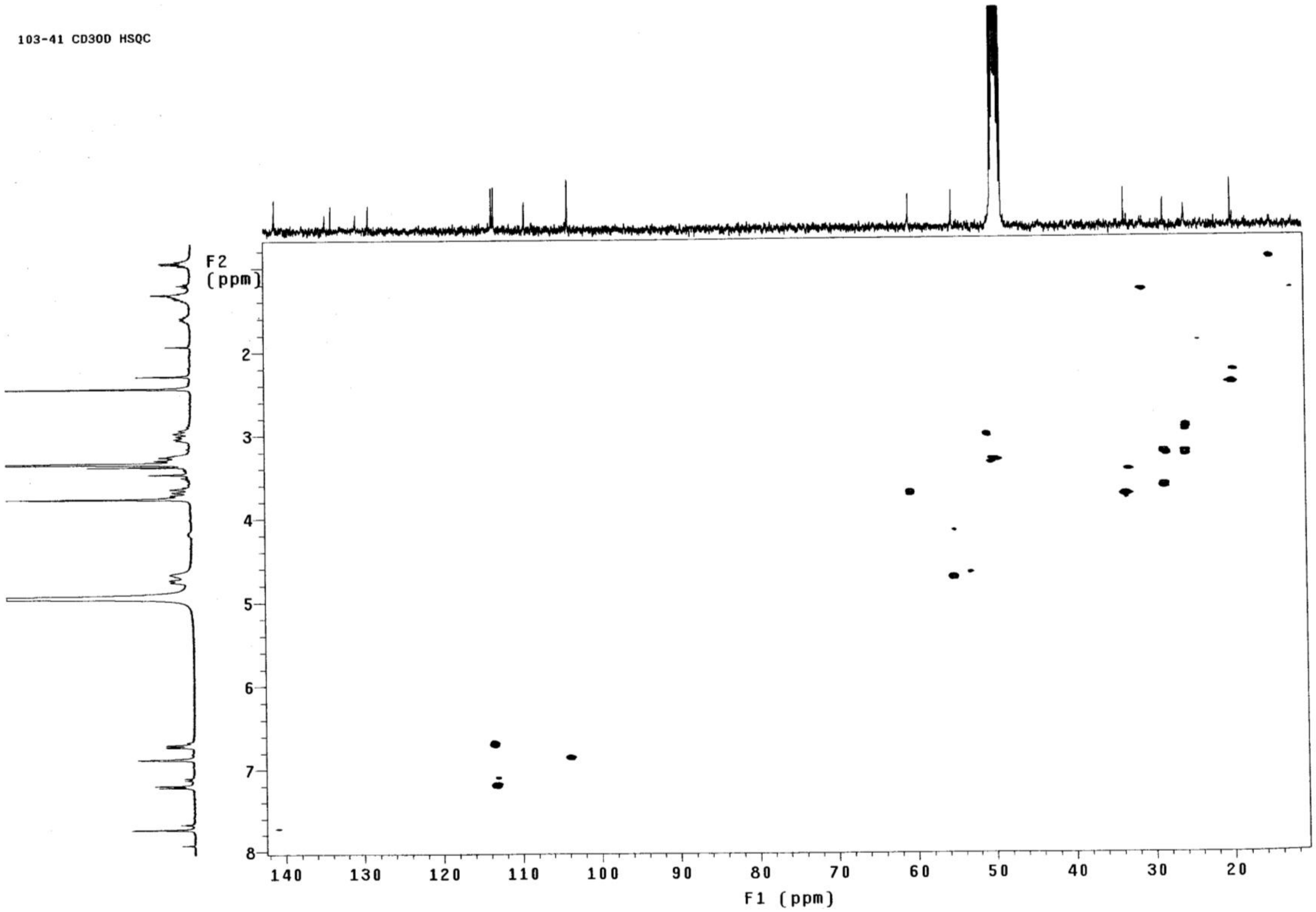

Figure S24. HMBC spectrum of Hainanerectamine C.

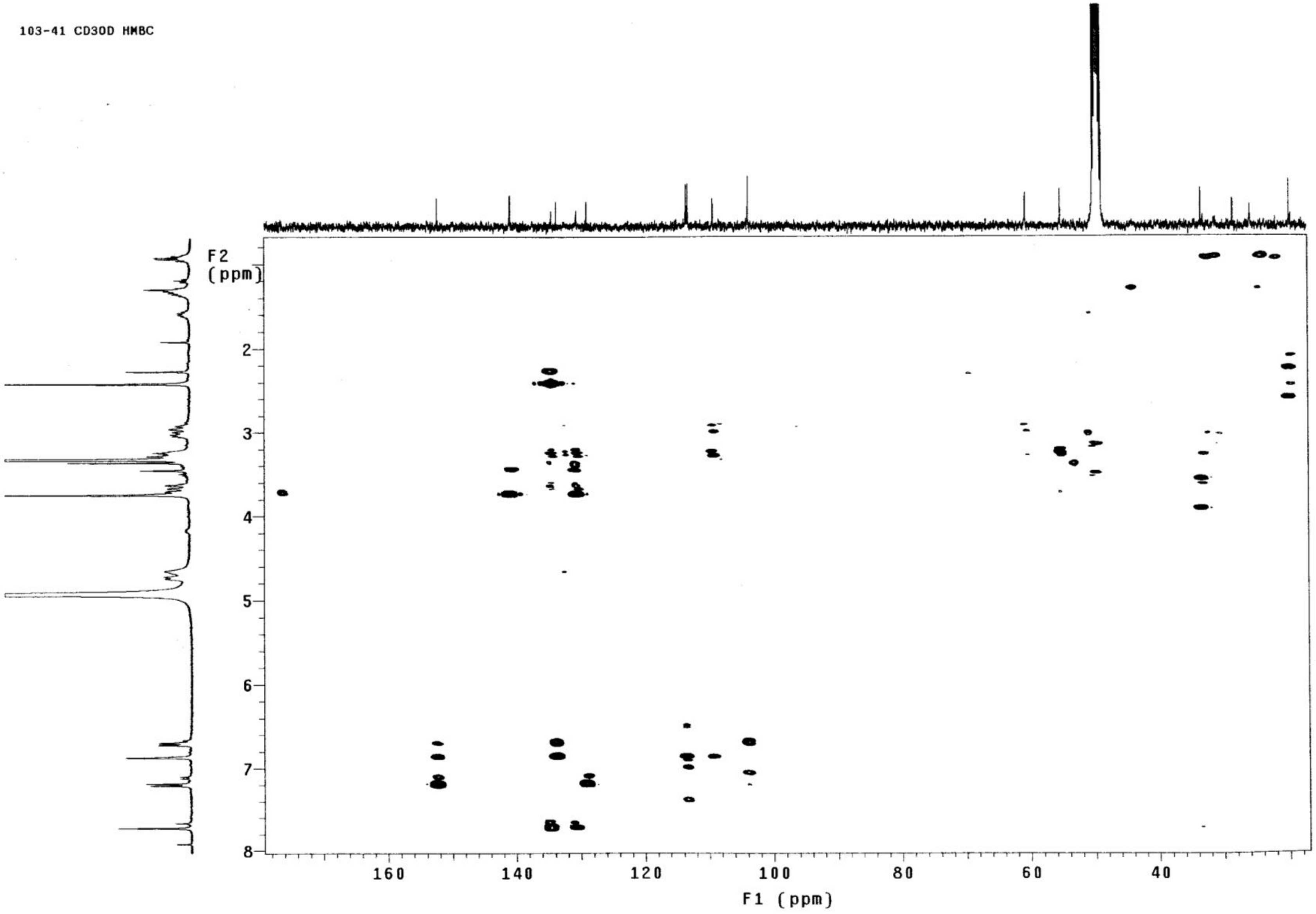

Figure S25. ^{1}H-^{1}H COSY spectrum of Hainanerectamine C.

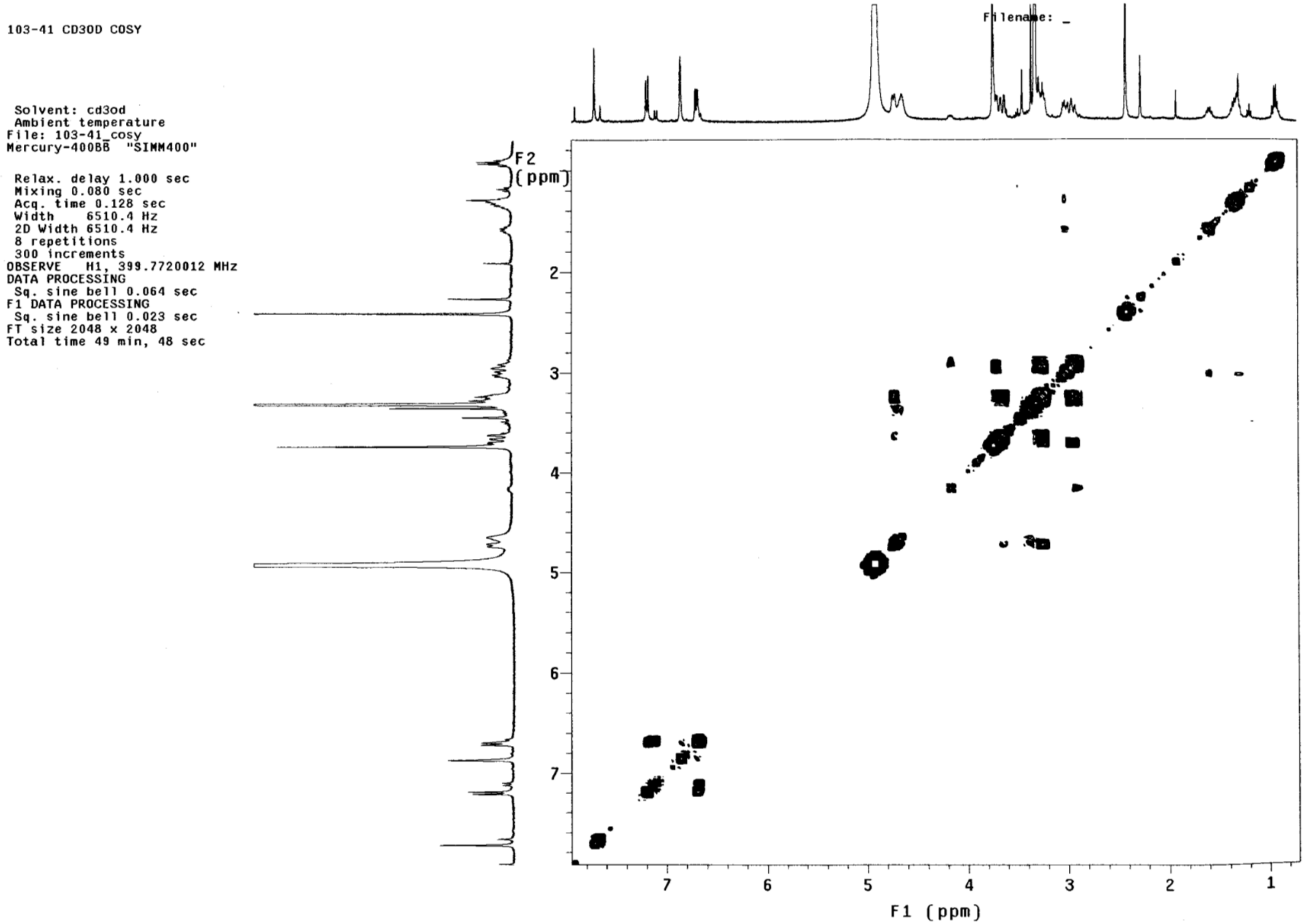

Figure S26. ROESY spectrum of Hainanerectamine C.

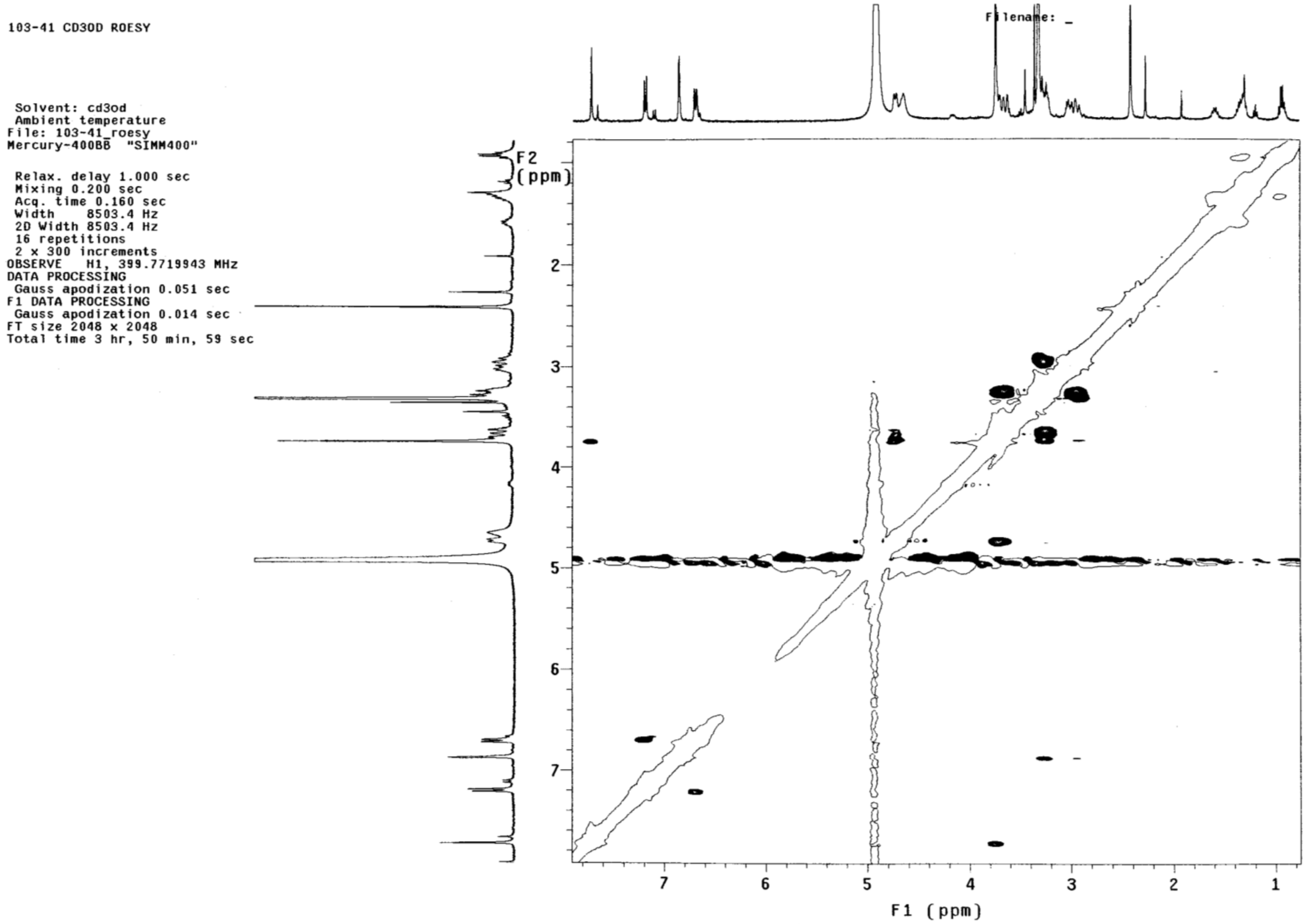

Figure S27. HRESI spectrum of Hainanerectamine C.

Elemental Composition Report

Page 1

Tolerance = 20.0 PPM / DBE: min = -1.5, max = 50.0
Isotope cluster parameters: Separation = 1.0 Abundance = 1.0%

Monoisotopic Mass, Odd and Even Electron Ions
36 formula(e) evaluated with 1 results within limits (up to 20 closest results for each mass)

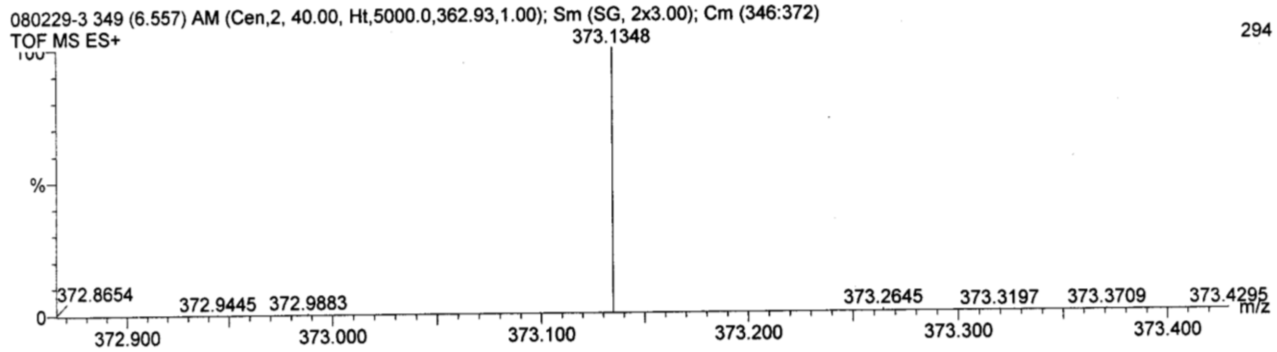

Minimum:	50.00				-1.5		
Maximum:	100.00		200.0	20.0	50.0		
Mass	RA	Calc. Mass	mDa	PPM	DBE	Score	Formula
373.1348	100.00	373.1334	1.4	3.7	10.5	1	C18 H21 N4 O3 S

Figure S28. IR spectrum of Hainanerectamine C.

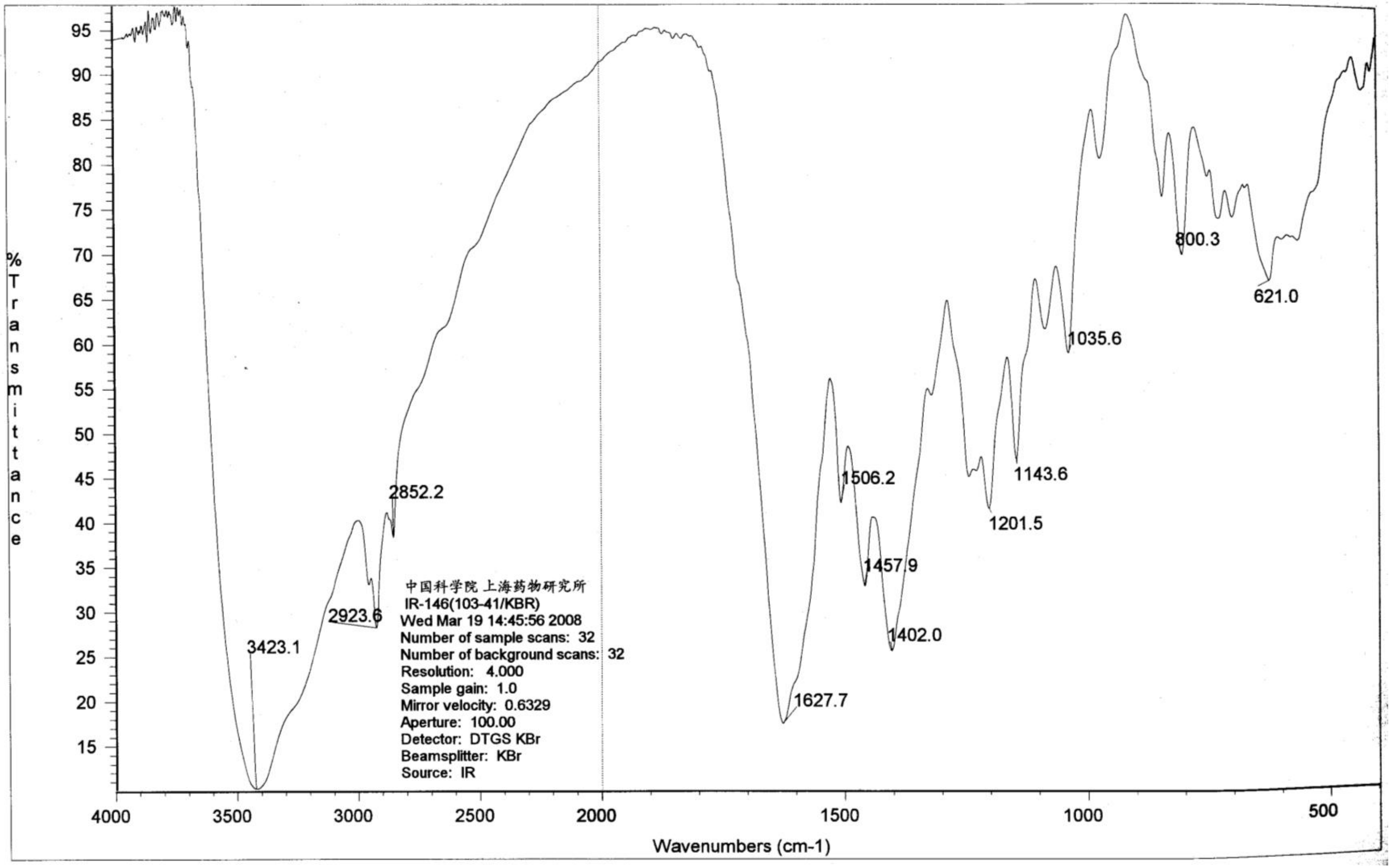

© 2014 by the authors; licensee MDPI, Basel, Switzerland. This article is an open access article distributed under the terms and conditions of the Creative Commons Attribution license (http://creativecommons.org/licenses/by/3.0/).
